# Crosstalk Between Intratumoral Microbes and Tumor Immunity: Implications for Tumor Therapy

**DOI:** 10.1002/cam4.71575

**Published:** 2026-01-26

**Authors:** Fengxue Li, Lili Qiao, Xinquan Liang, Yingying Zhang, Ning Liang, Jian Xie, Guodong Deng, Yuying Hao, Pingping Hu, Xue Wu, Fangjie Ding, Can Feng, Yiming Mu, Jiandong Zhang

**Affiliations:** ^1^ Shandong First Medical University & Shandong Academy of Medical Sciences Jinan China; ^2^ Department of Oncology, The First Affiliated Hospital of Shandong First Medical University & Shandong Provincial Qianfoshan Hospital Shandong Lung Cancer Institute Jinan China; ^3^ Clinical Medical College of Jining Medical University Jining Shandong China; ^4^ School of Clinical Medicine Shandong Second Medical University Weifang China

**Keywords:** Bacteria, Fungi, Intratumoral microbiota, tumor immune microenvironment, tumor therapy

## Abstract

**Background:**

Emerging studies indicate that microbes are present in tumor cells and immune cells. Intratumoral microbiota (ITM) constitute an important component of the tumor immune microenvironment (TIME) and have an important impact on tumor progression and treatment.

**Objective:**

Through the general elaboration of ITM represented by bacteria and fungi and the overall summary of their correlation with TIME, we aim to provide new ideas and perspectives for the application of ITM in tumor therapy by this review.

**Methods:**

This review conducted a literature search using the PubMed database, with no predefined restrictions on the publication time of the included literature. The search terms used included “intratumoral microbiota”, “intratumoral microbiome”, “intratumoral microbes”, “intratumoral microorganisms”, “tumor microbiota”, “tumor‐associated microbiota”, “tumor microbiome”, “tumor‐associated microbiome”, “tumor‐associated microbes”, “intratumoral bacteria”, “intratumoral fungi”, “cancer”, “tumor”, “tumor microenvironment”, “tumor immune microenvironment”, “microbial metabolites”, “application”, “immunotherapy”, “treatment” and “microbial‐based cancer therapy”. Relevant retrieved literature was screened, prioritizing studies that focused on the distribution characteristics of ITM across different tumors, the mechanistic insights and therapeutic potential.

**Results:**

Studies indicate that bacteria and fungi exhibit distinct distribution patterns in different tumors and interact with the TIME in complex ways, demonstrating either pro‐tumor or anti‐tumor effects. Proposed hypotheses for the underlying mechanisms include: (1) Antigenic immune responses, including those induced by bacterial peptides or cross‐immunity due to similarities between tumor and intratumoral microbial antigens; (2) The activation or inhibition of the function or infiltration of different immune cells; (3) Participating in pattern recognition receptor‐mediated signaling pathways; (4) Regulation of immune checkpoints or their inhibitors. ITM can also influence the efficacy of various tumor treatments, including chemotherapy, radiotherapy, and immunotherapy. Several microbial‐associated therapeutic approaches, such as engineered bacteria, have already entered clinical application. More treatment strategies are under investigation, although most current research remains at the level of establishing correlations between ITM and tumors, or is confined to preclinical experiments. Further exploration is required to establish causal relationships and achieve precise modulation.

**Conclusion:**

Given the significant role of ITM in tumor immunity, it may serve as a potential target for enhancing immunotherapy. Future research must shift its core focus from exploring correlations to intervening based on causality and achieving precise modulation for translational applications.

**Abbreviations**
3‐ICA: indole‐3‐carboxylic acid
A2AR: adenosine 2A receptor
AhR: aryl hydrocarbon receptor
A. sydowii: *Aspergillus sydowii*
BBP: benign biliary pathology
BCG: Bacillus Calmette‐Guerin
BLP: bacterial lipoproteins
CagA: cytotoxin‐associated gene A
CAR‐T: chimeric antigen receptor T‐cell
Ca‐type: Candida‐dominant
CCA: cholangiocarcinoma
CEACAM1: carcinoembryonic antigen‐related cell adhesion molecule 1
CRC: colorectal cancer
CREB: cAMP Response Element‐Binding Protein
CRT: calreticulin
CTL: cytotoxic T lymphocytes
DAMPs: danger‐associated molecular patterns
DCs: dendritic cells
EBV: Epstein–Barr virus
ECCA: extrahepatic cholangiocarcinoma
EcN:
*Escherichia coli* Nissle

*E. coli*

:
*Escherichia coli*

FMT: fecal microbiota transplantation
GC: gastric cancer
GI: gastrointestinal
GPR: G protein‐coupled receptors
HBL: hemolysin BL
HBV: hepatitis B virus
HCV: hepatitis C virus
HDACs: histone deacetylases
HIV: human immunodeficiency virus‐1
HLA: Human leukocyte antigens
HMBG1: high mobility group box 1
HPV: human papillomavirus

*H. pylori*

:
*Helicobacter pylori*

HTLV‐1: human T‐cell lymphotropic virus type 1
I3A: indole‐3‐aldehyde
ICD: immunogenic cell death
ICIs: immune checkpoint inhibitors
ILCs: innate lymphoid cells
iNOS: inducible nitric oxide synthase
ITM: intratumoral microbiota
ITS: internal transcribed spacer
KSHV: Kaposi's sarcoma‐associated herpesvirus
LUAD: lung adenocarcinoma
MBL: mannan‐binding lectin
MDSCs: myeloid‐derived suppressor cells
M‐MDSCs: mononuclear MDSCs
MSI‐H: microsatellite instability‐high
MTI: metronidazole‐triazole‐iodoacetamide
MTI‐FDU: metronidazole‐fluoropyridine nanoparticles
MYD88: myeloid differentiation primary response 88
NGS: next‐generation sequencing
NK: natural killer
NLRs: Nod‐liked receptors
NO: nitric oxide
Nod: nucleotide‐binding oligomerization domain
NPC: nasopharyngeal carcinoma
OMT: oral microbiota transplantation
OSCC: oral squamous cell carcinoma
OVs: oncolytic virus
PAMPs: pathogen‐associated molecular patterns
PDAC: pancreatic ductal adenocarcinoma
pDCs: plasmacytoid dendritic cells
PMN‐MDSCs: polymorphonuclear MDSCs
PRR: pattern recognition receptor
ROS: reactive oxygen species
Sa‐type: Saccharomyces‐dominant
SCFAs: short‐chain fatty acids
STAT3: signal transducer and activator of transcription
STING: stimulator of interferon genes
STS: soft tissue sarcomas
TANs: tumor‐associated neutrophils
TCGA: The Cancer Genome Atlas
TCR: T cell receptor
Th1: helper T cell type 1
TIME: tumor immune microenvironment
TLR‐5: Toll‐like receptor 5
TME: tumor microenvironment
Treg: regulatory T cells

## 1 Introduction

Advancements in technologies such as high‐throughput sequencing have significantly progressed our understanding of microbes. Microbial communities are widely present on various surfaces and in the gut of the human body, including bacteria, fungi, viruses, and other eukaryotic organisms. These microbes are involved in multiple physiological and pathological processes of the host and impinge on the risk of diseases, including cancer [1]. In recent years, the advent of metagenomic sequencing has confirmed the existence of intratumoral microbiota (ITM) and prioritized their research into tumors. Tumors, once thought to be sterile, are now known to harbor a variety of microbes, which have been shown to be closely associated with tumor development. Further research has identified ITM as important components of the tumor microenvironment (TME), playing vital roles in tumor initiation, colonization, metastasis, treatment, and prognosis [2]. Accumulating evidence has shown that ITM participate in tumor progression mainly by regulating the immune responses [3]. Thus, investigating the intricate relationship between ITM and tumors, as well as their interactions with the tumor immune microenvironment (TIME), is significant for understanding tumor development. This review aims to explore the interplay between ITM with the TIME and their potential in tumor therapy.

## 2 Discovery and Characteristics of ITM

The human body hosts a vast array of microbial communities, including bacteria, viruses, fungi, and other eukaryotic organisms [1], residing in the oral cavity, gastrointestinal (GI) tract, reproductive organs, and skin [4]. There are some organisms officially recognized as unique causes of human cancer, such as Epstein–Barr virus (EBV), hepatitis B virus (HBV), hepatitis C virus (HCV), Kaposi's sarcoma‐associated herpesvirus (KSHV), human immunodeficiency virus‐1 (HIV), human papillomavirus (HPV), human T‐cell lymphotropic virus type 1 (HTLV‐1), and *Helicobacter pylori* ( *H. pylori* ). These microbes promote tumor progression through various mechanisms, inducing B‐cell differentiation, disrupting cell cycle regulation, immune overactivation (in EBV, HBV, HCV, and HIV infections), T cell dysregulation (in EBV and HTLV infections), and directly inducing tumorigenesis in liver cancer and Kaposi's sarcoma by hepatitis viruses and KSHV, respectively [5].

**FIGURE 1:**
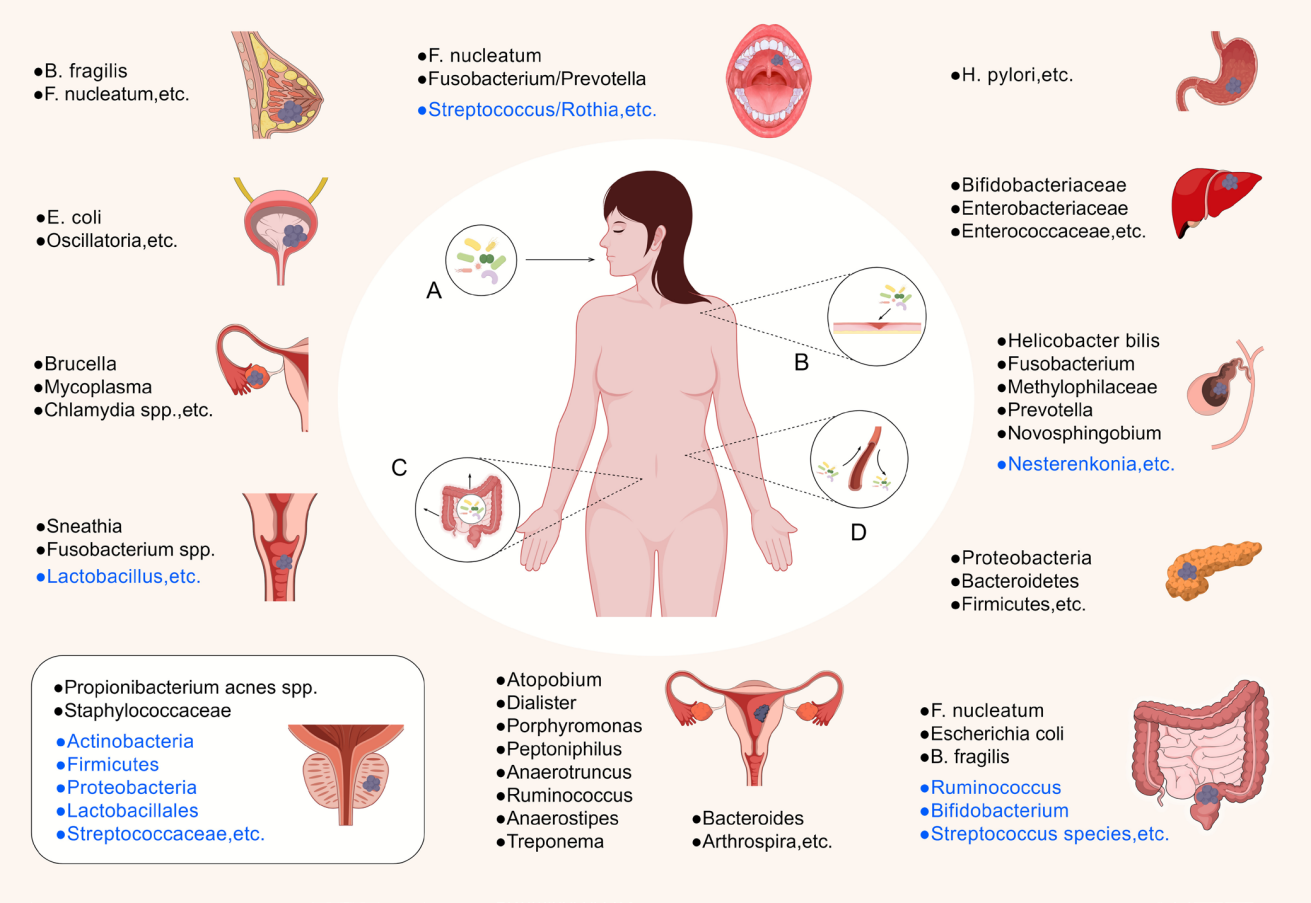
Sources and distribution of the intratumoral microbiota (ITM). The central part presents several possible sources of ITM: (A) The oral cavity is considered one of the origins of ITM. (B) The microbes can originate from the external environment and enter the body by breaking through the body's defense barriers, such as wound infections. (C) The intestine is one of the sources of ITM. (D) Bacteria from distant or adjacent organs/tissues spread to tumor tissues via blood or other physical channels. And, the peripheral section shows the distribution of intratumoral bacteria in various types of cancer, including oral cancer, bladder cancer, digestive system tumors, and reproductive system tumors. Furthermore, the bacteria are distinguished by different colors including black (increased bacteria in tumors) and blue (decreased bacteria in tumors). ITM, intratumoral microbiota.

Organs and tissues, such as the lungs, prostate, bladder, breast, liver, and pancreas, which were once thought to be sterile, are now identified as potential reservoirs of low‐biomass microbial populations with the advent of next‐generation sequencing (NGS) [5]. Similarly, tumors have long been considered sterile environments, and the discovery of intratumoral microbiota (ITM) has overturned this view. Using 16S rRNA gene sequencing technology, bacterial DNAs have been detected in various types of tumor tissues, including human solid tumors. Distribution of these bacterial 16S rRNA suggests that intratumoral bacteria are primarily located within cancer cells and immune cells [6]. There are several different sources of ITM (Figure 1). These microbes can originate from the external environment such as infections or may be transferred from other parts of the body's microbes to the tumor microenvironment (TME) due to dysbiosis or other reasons, including microbes of adjacent tissues and distant organs, or they may be indigenous. Bacteria from distant organs can spread to tumor tissues via blood or other physical channels. Common sources of microbes within the body are the gut microbes and the oral microbes. The gut is the main organ colonized by commensal microbes [7]. Clinical data have revealed that microbes' expression overlaps between tumor tissues and fecal samples, indicating that the intestine is the source of ITM [8, 9]. The microbes might be translocated from the intestinal tract into other organs via the blood circulation and/or bile, hepatic, and pancreatic ducts [10]. Similarly, the oral cavity also has been regarded as the origin of the ITM. As the entry portal for the GI tract, the oral cavity is connected to the respiratory tract, and microbiota residing in the oral cavity can disseminate into the respiratory and GI tracts [11], and consequently, oral microbes may have an impact on cancer in these areas. For instance, the esophageal microbiota exhibits a resemblance to the oral microbiota, including Firmicutes, Bacteroides, Actinobacteria, Proteobacteria, Fusobacteria, and TM7, suggesting the translocation of microbial populations from the oral cavity to the esophageal region [12]. The translocation of oral bacteria to the respiratory tract and GI organs might be achieved via the bloodstream and the digestive tract [13].

Nejman, D. et al. studied the ITM in several solid tumors of the body, revealing a phenomenon that the types and abundances of ITM vary significantly between individuals and tumor types [6]. Several factors influence ITM colonization, including tumor risk factors and race, and the composition of ITM also shows significant correlations with different tumor biology [14, 15]. Diet is also suggested to be a potential factor affecting the composition of the ITM [16]. Hypoxia and immune suppression in the TME could promote microbial growth, which may explain the migration of microbes from noncancerous to cancerous tissues [17]. Different tumor types have different angiogenesis and oxygen levels, endocytosis and micropinocytosis activities, and microbial sources in the surrounding tissue [18]. These factors collectively may determine the composition of the ITM and form tumor type‐specific signatures. The TME is often immunosuppressive, which could protect bacteria from clearance by the immune system, resulting in different immune subtypes potentially favoring different bacteria [19, 20]. ITM can affect tumor cell proliferation and apoptosis, regulate the TME and the immune responses to promote tumor development. Their metabolites or metabolic products can also have a range of effects on the TME. In preclinical models, microbial metabolites can regulate phenotypes of tumor somatic mutations and modulate the efficacy of immune checkpoint inhibitors (ICIs) [21]. Thus, ITM and their metabolites are also considered components of the TME.

## 3 Distribution of ITM

### 3.1 Bacteria

Bacterial components are the most abundant in intratumoral microbiota (ITM). In the tumor microenvironment (TME), there is one bacterial cell for every 147 cancer cells [1]. Bacteria are currently among the most studied types of ITM, with numerous experiments confirming their presence. The distribution of the ITM is not random but highly organized [22]. The diversity of ITM in cancer tissues is generally lower than that in corresponding normal tissues [23]. The relative specificity of ITM also offers new approaches for tumor diagnosis. The symbiotic microbes comprise bacteria, archaea, viruses, fungi, and other eukaryotic organisms. Symbiotic microbes inhabit all mucosal barrier surfaces, with the most abundant populations residing in the gut [1]. Certain bacterial components of the gut microbes can drive tumorigenesis in GI tissues. For example, experimental evidence supports a close link between *H. pylori* and the progression of atrophic gastritis, metaplasia, atypical hyperplasia, and gastric cancer (GC) [24]. These results suggest that the gut microbes can modulate the ITM, with these changes potentially being partly due to the direct transfer of gut bacteria, but more importantly, by altering the composition of bacteria within the tumor.

In colorectal tumors, the populations of *F. nucleatum* [25], *Escherichia coli* ( *E. coli* ), *B. fragilis* [26] are increased, whereas those of Ruminococcus, Bifidobacterium, and Streptococcus species are decreased [8]. In tumor samples from patients with cholangiocarcinoma (CCA), Bifidobacteriaceae, Enterobacteriaceae, and Enterococcaceae are enriched [27]. In patients with extrahepatic cholangiocarcinoma (ECCA), compared to those with benign biliary pathology (BBP), the population of Nesterenkonia is decreased, while the populations of *Helicobacter bilis* , Fusobacterium, Methylophilaceae, Prevotella, Novosphingobium, Actinomyces, and *H. pylori* are increased [28]. In hepatocellular carcinoma tissues, the abundance of Helicobacter species is relatively high [29]. In GC, *H. pylori* is one of the first bacterial species to be definitively linked to the onset of tumorigenesis [30]. In tissues from mouse models and humans with pancreatic cancer, Proteobacteria, Bacteroidetes, and Firmicutes are abundant [10]. In oral cancer, abnormal bacterial abundance such as increased *F. nucleatum* [31] and Fusobacterium/Prevotella [32] and decreased Streptococcus/Rothia [33] in the oral cavity might be a risk factor. In addition to the digestive system, there are also many related studies on the microbes in tumors of other systems. In freshly resected prostate tissues, over 40 unique bacterial genera have been identified [34]. The abundance of Staphylococcaceae [35] and *Propionibacterium acnes* spp. [36] is increased, and the biomass of Actinobacteria, Firmicutes, Proteobacteria, Lactobacillales, and Streptococcaceae is decreased [35]. In bladder cancer, the increased *E. coli* , the butyrate‐producing bacterium SM4/1, and Oscillatoria might be associated with poor prognosis [37]. In endometrial cancer, the increased abundance of Atopobium, Dialister, Porphyromonas, Peptoniphilus, Anaerotruncus, Ruminococcus, Anaerostipes, Treponema, Bacteroides, and Arthrospira promotes carcinogenesis [38]. In samples from patients with ovarian cancer, the prevalence of Brucella, Mycoplasma and Chlamydia spp. has been confirmed [39]. In cervical cancer, which is the most prevalent malignancy associated with HPV, the increased abundance of Sneathia [40] and Fusobacterium spp. [41], and decreased Lactobacillus biomass promotes the oncogenesis [40]. Common *B. fragilis* [42] and *F. nucleatum* [43] also contribute to breast cancer progression.

Tumor‐type specific microbes are primarily composed of bacteria (Figure 1). Common intratumoral bacteria in research include *Klebsiella pneumoniae* , *Enterobacter cloacae* , *Citrobacter freundii* , *Enterobacter asburiae* , and Fusobacterium [6]. It is currently unclear whether these microbes represent a predetermined ecological niche or a transient random colonization [5]. Each microbial niche may mediate community‐level interactions by altering the microbe configuration, also known as dysbiosis. The impact of specific ecological niches on cancer can be exemplified by the influence of the oral microbes on cancers of the oral cavity (oral squamous cell carcinoma [OSCC]), colon (colorectal cancer [CRC]), and pancreas (pancreatic ductal adenocarcinoma [PDAC]) [44]. Although the number of bacterial cells in the human body is similar to that of human cells, the genetic diversity of bacteria is 100 times that of human cells. Moreover, the mechanisms and metabolic capabilities encoded by bacteria not only affect their own microbial niches but also influence the tissue specificity and immune cell functions of the host [45]. Within tumors, bacteria can regulate both the intrinsic characteristics of cancer cells and their external environment, thereby enhancing the ability of cancer cells and paving the way for cancer metastasis [18]. Besides directly modulating cancer cells, intratumoral bacteria are also important inflammatory mediators that can shape the specific microenvironment around cancer cells, thereby indirectly promoting cancer metastasis. Intratumoral bacteria can inhibit immune responses, thereby promoting tumor development and metastasis. At the same time, bacteria can also inhibit tumor progression. Intratumoral bacteria can trigger antitumor immunity. Probiotics can significantly enhance antitumor immunity against melanoma lung metastasis in mice [46]. Additionally, intratumoral injection of Bifidobacterium can stimulate relevant pathways, increase dendritic cells (DCs) numbers, and enhance anti‐CD47‐based immunotherapy [47]. Overgrowth of intratumoral bacteria induces tumor regression through both direct and indirect mechanisms. Salmonella spp., for instance, induce autophagy to directly kill tumor cells by producing toxins and depleting nutrients. Salmonella flagellin signals through Toll‐like receptor 5 (TLR5) to inhibit tumor cell proliferation. Indirectly, bacterial infections activate complex immune cell populations within the TME to enhance tumor surveillance and clearance [48]. In summary, the presence of bacteria within tumors has a complex impact on tumor development and progression. It can promote tumor development by directly damaging DNA, and it can also inhibit tumor progression by activating the immune system of the host. Furthermore, the metabolism of bacteria within tumors is closely related to the metabolism of the tumor itself. The specific mechanisms involved remain to be further studied.

### 3.2 Fungi

In addition to bacteria, fungi are another group of intratumoral microbiota (ITM). Considering the multifaceted roles of microbes in cancer, viewing microbes as just bacterial is limited. Fungi have also been detected in CRC, lung cancer, prostate cancer, GC, and skin cancer [22, 35]. In certain types of tumors, different fungal communities have been found to contribute to carcinogenesis [49]. Several new studies, using internal transcribed spacer (ITS) sequencing and whole genome sequencing methods or others to analyze the sequencing data from the Weizmann cohort or The Cancer Genome Atlas (TCGA) cohort, present a comprehensive picture of the tumor‐associated microbes from a variety of human cancers [50, 51]. These studies reveal that fungi, although in low abundance, are ubiquitous across all major human cancers and that specific microbe types can be predictive of survival.

The fungi are less populous than the bacteriome but can still greatly affect human health. Similar to the characteristics of the bacteriome, the differences in fungal components among individuals within a population result from the complex interplay of multiple factors, including geographical location, gender, age, ethnicity, diet, and lifestyle. Narunsky‐Haziza et al. characterized the cancer microbes within 17,401 patient‐derived tissue, blood, and plasma samples across 35 cancer types from four independent cohorts [50]. Subsequent researchers have built upon this foundation by utilizing data from the Weizmann cohort and TCGA cohort, aiming to define a variety of cancer‐associated fungal characteristics and further employing various staining techniques to visualize fungi within tumors [49, 50]. The research data indicate that fungi are universally present across major human cancers, with cancer‐specific compositions. Histological staining of tissue revealed the intratumoral presence of fungi predominantly localized to cancer cells and macrophages, similar to bacteria [52]. Significant differences in fungal richness were observed among different cancer types [49]. Narunsky‐Haziza et al. identified a variety of intratumoral fungi, including *Saccharomyces cerevisiae* , Malassezia restricta, *Candida albicans* , Malassezia globosa, and Blastomyces gilchristii [50]. This study uncovered the presence of low‐abundance, cancer type‐specific microbes. Additionally, Narunsky‐Haziza et al. analyzed the plasma microbes; significant differences were found between cancer and controls. A signature of circulating fungal DNA from 20 different fungi that potentially can be used to distinguish between pan‐cancer and healthy individuals was identified, indicating the practicality of using the microbes in cancer diagnostics, even in patients with early‐stage disease [50].

Fungi are distributed differently in diverse types of tumors. Fungi classes Malasseziomycetes, Saccharomycetes, Diothideomycetes, Sordariomycetes, and the genus Candida are significantly enriched in a variety of cancer types, while some specific fungi were only found at particular cancer sites [49]. An independent study by Dohlman et al. reported a pan‐cancer analysis of anatomically distinct body sites and identified a variety of tumor‐specific microbes [51]. And metagenomic analyses proved that fungi including Blastomyces gilchristii, *Candida albicans* , Malassezia globosa, Malassezia restricta, and *Saccharomyces cerevisiae* were present in various human cancer types [52]. Fungal DNA was particularly abundant in tissues of the head and neck, colon, rectum, and stomach, while it was less prevalent in the esophagus and negligible in brain tissue. The fungal composition was more similar in head and neck, colorectal, and colon cancers, as was the case for gastric and esophageal cancers, while non‐GI cancers had a fungal composition distinct from GI cancers. Specifically, various Candida and *Saccharomyces cerevisiae* were enriched in GI cancer samples, while Bipolaris and Malassezia were enriched in lung and breast cancer samples, respectively [51]. This study further revealed that the relative abundance of *Candida albicans* and *Saccharomyces cerevisiae* differed in GI tumors, suggesting that GI cancers could be categorized into Candida and Saccharomyces‐associated tumors. Candida‐dominant (Ca‐type) and Saccharomyces‐dominant (Sa‐type) tumors exhibited differences in gene expression, treatment, and survival times [51]. Blastomyces was very prevalent in lung tumor tissues, while in GC, a higher abundance of Candida was observed with higher expression of proinflammatory immune pathways. Furthermore, Candida could also interact with bacterial species and positively correlated with Lactobacillus spp., whereas it was negatively associated with *H. pylori* [49]. In CRC, Candida was not only predictive of advanced disease and metastasis but also linked with a reduction in cellular adhesion [49]. The fungal abundance in PDAC is approximately 3000 times that of normal, with a notable enrichment of the Malassezia genus in its community [22]. Alam and colleagues also demonstrated that pancreatic cancer was infiltrated by Alternaria and Malassezia, two well‐known fungal genera present in the intestine [53].

## 4 Roles of ITM in the TIME

**FIGURE 2:**
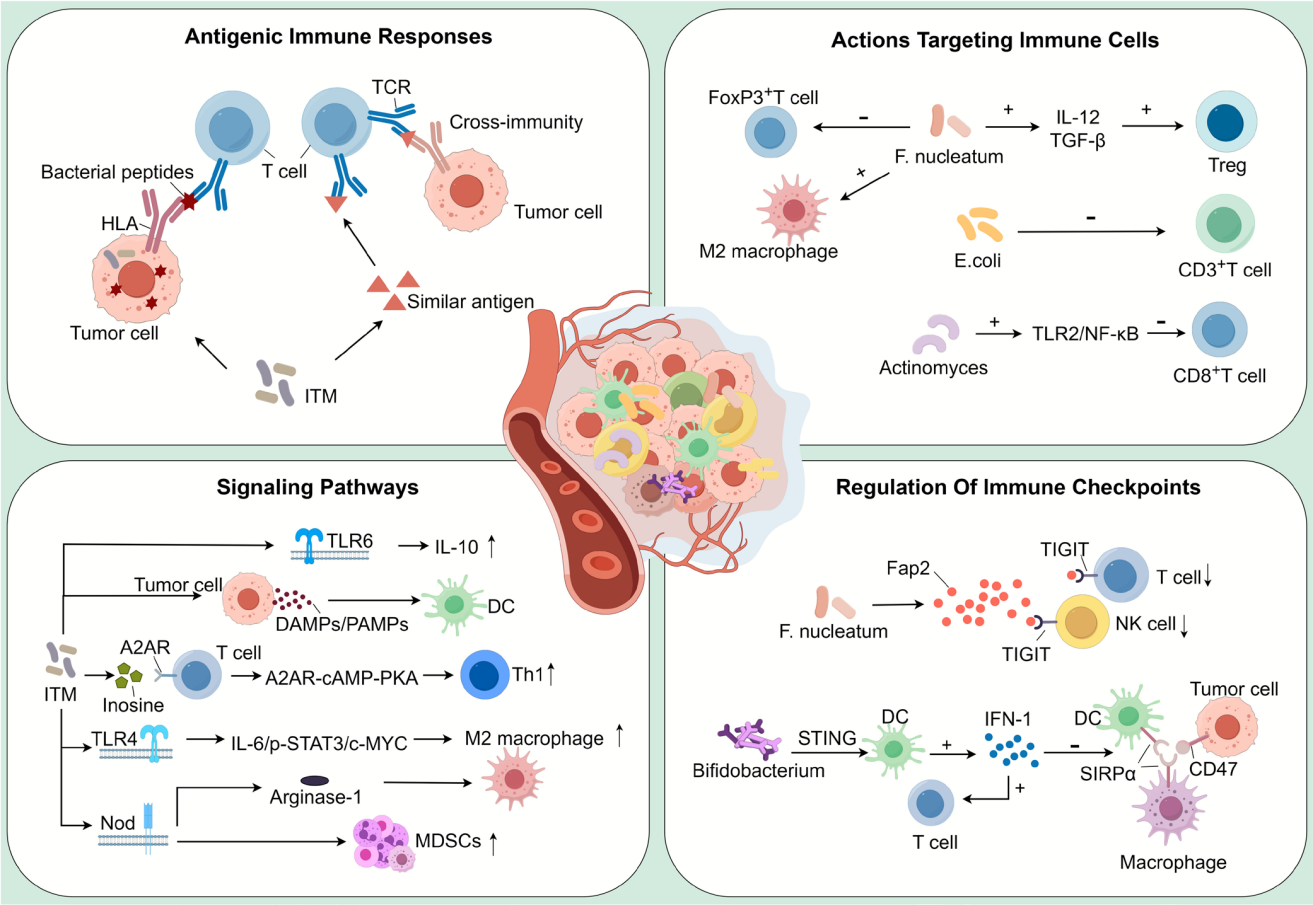
Intratumoral microbiota (ITM) can regulate the tumor immune microenvironment (TIME) by participating in the functions or related signaling pathways of immune cells. (A) Antigenic immune responses: ITM can enhance tumor immunogenicity by generating bacterial peptides recognized by HLA or antigens similar to those of tumors; (B) actions targeting immune cells: ITM can promote or suppress the infiltration of different immune cells within the TIME to regulate immune responses. For instance, *Fusobacterium nucleatum* promotes M2 macrophages and Treg cells while inhibiting FoxP3^+^T cell infiltration; *Escherichia coli* suppresses CD3^+^T cell infiltration; Actinobacteria reduce CD8^+^T cell infiltration; (C) signaling pathways: ITM participates in various signaling pathways to regulate the TIME, including the activation of TLRs, Nod‐like receptors, A2AR, etc.; (D) regulation of immune checkpoints: ITM can regulate immune checkpoints to influence immune responses. For example, *Fusobacterium nucleatum* interacts with TIGIT via Fap2 to inhibit the activity of NK cells and cytotoxic T cells, promoting immune evasion; Bifidobacterium enhances antitumor immunity by blocking CD47 through activation of the STING and interferon pathways. A2AR, adenosine 2A receptor; DAMPs, danger‐associated molecular patterns; DC, dendritic cell; HLA, human leukocyte antigens; ICD, immunogenic cell death; MDSCs, myeloid‐derived suppressor cells; NK, natural killer; Nod, nucleotide‐binding oligomerization domain; PAMPs, pathogen‐associated molecular patterns; PRR, pattern recognition receptor; STAT, signal transducer and activator of transcription; STING, stimulator of interferon genes; TCR, T‐cell receptor; Th, helper T cell; TLR, Toll‐like receptor; TME, tumor microenvironment; Treg, regulatory T cells.

The primary immune cells in the tumor immune microenvironment (TIME) can be roughly categorized into two groups based on their tumor‐promoting or tumor‐suppressing effects: One group consists of traditionally recognized tumor‐suppressing cells, such as activated CD8^+^T cells, activated CD4^+^T cells, helper T cell type 1 (Th1), DCs, natural killer T (NK) cells, M1‐like macrophages, and neutrophils, etc.; the other group comprises tumor‐promoting or immunosuppressive cells, such as M2‐like macrophages, regulatory T cells (Treg), Th17, myeloid‐derived suppressor cells (MDSCs), and inactive CD8^+^T cells [17, 54]. The immunosuppressive tumor microenvironment (TME) can weaken the immune response during tumor development and metastasis. For instance, MDSCs, composed of polymorphonuclear MDSCs (PMN‐MDSCs) and mononuclear MDSCs (M‐MDSCs), inhibit T cell proliferation and activation by generating high levels of arginase 1, reactive oxygen species (ROS), and nitric oxide (NO), thereby promoting tumor angiogenesis. Macrophages exist in two distinct polarization states: M1‐like macrophages have antitumor activity, while M2‐like cells have tumor‐promoting and immunosuppressive properties. M2 macrophages express arginase 1, which processes and consumes L‐arginine, a key factor for T‐cell function. Meanwhile, Tregs can induce an immunosuppressive phenotype that fosters tumor progression [55]. Several cross‐sectional studies in recent years have observed the correlation between the intratumoral microbiota (ITM) and the TIME [56, 57]. In addition to the observations from cross‐sectional studies, some preclinical experiments have also analyzed the correlation between these two entities. For instance, using a breast cancer mouse model, Lishay Parhi and colleagues discovered that *F. nucleatum* specifically accumulates within tumors, reducing the infiltration of CD4^+^T cells and CD8^+^T cells [43]. Some studies have proposed possible hypotheses about the mechanisms involved (Figure 2), including: (1) Antigenic immune responses, including those induced by bacterial peptides or cross‐immunity due to similarities between tumor and intratumoral microbial antigens; (2) Actions targeting immune cells, including the activation or suppression of different immune cell functions or infiltration by the ITM, or the induction of immunogenic cell death (ICD); (3) ITM participating in pattern recognition receptor (PRR)‐mediated signaling pathways; (4) Regulation of immune checkpoints or their inhibitors by the ITM [17, 58]. Research on the related pathway mechanisms is crucial for further exploration of the role of the ITM in tumor progression, and it is also a necessary condition for the future application of the ITM in cancer diagnosis, treatment, and prognosis. Below, we will briefly elaborate on the related mechanisms through several specific tumors and their associated ITM (Tables 1 and 2).

**TABLE 1:** Influence and role of intratumoral bacteria on the TIME of different tumors.

Tumor	ITM	Associated immune cells or signaling molecules	Effects	References
CRC	*F. nucleatum*	Inversely proportional to the density of FoxP3^+^T cells and proportional to the proportion of M2‐like TAMs.	Promote	[59]
	*F. nucleatum*	Recruit CD11b^+^myeloid cells to the TIME.	Promote	[60]
	*F. nucleatum*	Induce the release of IL‐12 and TGF‐β, thereby increasing the number of Tregs.	Promote	[61]
	*F. nucleatum*	Upregulate the IL‐6/p‐STAT3/c‐MYC pathway then leading to M2‐like TAM polarization.	Promote	[62]
	*F. nucleatum*	Inhibit the activity of NK cells and cytotoxic T cells through the interaction between Fap2 and TIGIT, or between Fap2 and CEACAM1.	Promote	[63, 64]
	*E. coli*	Reduction of CD3^+^ and CD8^+^T cells, resistance to anti‐mouse PD‐1 immunotherapy.	Promote	[57]
	Actinomyces	Activate the TLR2/NF‐κB pathway and reduce CD8^+^T cells.	Promote	[65]
	Ruminococcaceae	Associated with M1‐like TAMs.	Associated	[61]
	Faecalibacterium	Associated with M1‐like TAMs.	Associated	[61]
	Eubacterium	Associated with M1‐like TAMs.	Associated	[61]
	Bacteroides	Associated with M1‐like TAMs.	Associated	[61]
	OVs	Target the TME and lyse tumor cells, and release tumor antigens, DAMPs, and PAMPs.	Inhibit	[66, 67]
	Lactobacillus	Trigger IL‐10 expression in a TLR‐6‐dependent manner.	Inhibit	[68]
	*Bifidobacterium pseudolongum*	Secret inosine, initiate the A2AR‐cAMP‐PKA pathway, and induce differentiation of naïve T cells into CD4^+^Th1.	Inhibit	[69]
	Pathogenic Neisseria	Bind to CEACAM1.	Associated	[17]
Melanoma	*F. nucleatum*	HLA‐I presents the bacterial peptide antigens, activate CD4^+^Tcell and CD8^+^Tcell.	Inhibit	[70]
	*Staphylococcus aureus*	HLA‐I presents the bacterial peptide antigens, activate CD4^+^Tcell and CD8^+^Tcell.	Inhibit	[70]
	*Staphylococcus capitis*	HLA‐I presents the bacterial peptide antigens, activate CD4^+^Tcell and CD8^+^Tcell.	Inhibit	[70]
	*Bifidobacterium breve*	Induce the production of SVY‐specific T cells with the epitope SVY.	Inhibit	[71]
	Lachnoclostridium	Promote CD8^+^Tcell infiltration by modulate chemokines including CXCL9, CXCL10, and CCL5.	Inhibit	[72]
	Gelidibacter	Promote CD8^+^Tcell infiltration by modulate chemokines including CXCL9, CXCL10, and CCL5.	Inhibit	[72]
	Flammeovirga	Promote CD8^+^Tcell infiltration by modulate chemokines including CXCL9, CXCL10, and CCL5.	Inhibit	[72]
	Acinetobacter	Promote CD8^+^Tcell infiltration by modulate chemokines including CXCL9, CXCL10, and CCL5.	Inhibit	[72]
	Tropobacter	Promote CD8^+^Tcell infiltration by modulate chemokines including CXCL9, CXCL10, and CCL5.	Inhibit	[72]
	Algibacter	Negative correlation with CD8^+^T cells.	Associated	[72]
	Epilithonimonas	Negative correlation with CD8^+^T cells.	Associated	[72]
Lung cancer	*E. coli*	Proteins that cross‐react with EBV and *E. coli* are overexpressed, and this cross‐reactivity may lead to virus‐specific T‐cell infiltration TIME.	Associated	[73]
	Staphylococcus	Stimulate myeloid cells to secrete IL‐1β and IL‐23 and induce Vγ6^+^Vδ1^+^γδT cells to proliferate and secrete IL‐17.	Promote	[74]
	Streptococcus	Stimulate myeloid cells to secrete IL‐1β and IL‐23 and induce Vγ6^+^Vδ1^+^γδT cells to proliferate and secrete IL‐17.	Promote	[74]
	Lactobacillus	Stimulate myeloid cells to secrete IL‐1β and IL‐23 and induce Vγ6^+^Vδ1^+^γδT cells to proliferate and secrete IL‐17.	Promote	[74]
	Pasteurellaceae	Stimulate myeloid cells to secrete IL‐1β and IL‐23 and induce Vγ6^+^Vδ1^+^γδT cells to proliferate and secrete IL‐17.	Promote	[74]
	Lachnoclostridium	Positively correlated with M1 macrophages.	Associated	[75]
GC	Stenotrophomonas	Positively correlated with the BDCA2^+^pDCs and Foxp3^+^Tregs.	Promote	[76]
	Selenomonas	Positively correlated with the BDCA2^+^pDCs and Foxp3^+^Tregs.	Promote	[76]
	*H. pylori*	Its HopQ interacts with CEACAM1, leading to inhibition of immune cell.	Promote	[77]
PDAC	ITM	Reduce CD8^+^T cells and lower immune reactivity via the IFN pathway.	Promote	[58, 78]
	ITM	Activate TLRs in monocytic cells to induce M2‐like TAM differentiation.	Promote	[10]
	Lactobacilli	Increase AhR transcriptional responses, promoting an immunosuppressive TME.	Promote	[79]
Bladder Cancer	ITM	Associated with Treg.	Associated	[80]
CCA	ITM	Induce hepatocytes to express CXCL1 and recruit CXCR2^+^ polymorphonuclear MDSCs.	Promote	[81]
Skin cancer	ITM	Upregulate a protein called high mobility group box 1 and trigger inflammation and skin cancer.	Promote	[82]
Liver cancer	ITM	The butyrate produced inhibits HDAC, increasing CD8^+^T cell.	Inhibit	[17]
NPC	Shewanella	Associated with a high degree of neutrophil infiltration.	Associated	[83]
	Rhodobacter	Associated with a high degree of neutrophil infiltration.	Associated	[83]
	Altererythrobacter	Associated with a high degree of neutrophil infiltration.	Associated	[83]
STS	HHV‐ 6	Positively correlated with NK cells.	Associated	[84]
	Respirovirus	Positively correlated with NK cells.	Associated	[84]

Abbreviations: A2AR, adenosine 2A receptor; CCA, cholangiocarcinoma; CEACAM1, carcinoembryonic antigen‐related cell adhesion molecule; CRC, colorectal cancer; NK, natural killer T; DAMPs, danger‐associated molecular patterns; *E. coli* : *Escherichia coli;* EBV, Epstein–Barr virus; GC, Gastric cancer; *H. pylori* , *Helicobacter pylori* ; HDAC, histone deacetylase; ITM, intratumoral microbiota; MDSCs, myeloid‐derived suppressor cells; NPC, nasopharyngeal carcinoma; Ovs, oncolytic viruses; PAMPs, pathogen‐associated molecular patterns; PDAC, pancreatic ductal adenocarcinoma; pDCs, plasmacytoid dendritic cells; STS, soft tissue sarcomas; TAMs, tumor‐associated macrophages; TIME, tumor immune microenvironment.

**TABLE 2:** Influence and role of fungi on the TIME of different tumors.

Tumors	Fungi	Associated immune cells or signaling molecules	Effects	References
GC	Candida	Associated with genes related to cytokine interactions, host immunity, and inflammation including IL1A, IL1B, IL6, IL8, CXCL1, CXCL2, and IL17C.	Associated	[51]
GC	Candida	Associated with genes involved in cytosolic DNA sensing, TLR signaling, and Nod‐like receptor signaling.	Associated	[51]
CRC	Candida	Down‐regulate tumor suppressor genes and genes regulating the cell adhesion pathway including PTK2B, CDKN2C, and NET1.	Associated	[51]
Head and neck cancers	Candida	Associated with low expression of tumor suppressors TP53 and CDKN2A as well as fibronectin.	Associated	[51]
Colon tumors	Candida	Trigger glycolysis in macrophages and the secretion of IL‐7, thereby inducing RORγt^+^ILCs to produce IL‐22 via AhR and STAT3 pathways.	Promote	[85]
PDAC	Malassezia	Adhere to MBL to activate the complement cascade and cleave C3 into C3a and C3b.	Promote	[86, 87]
PDAC	Fungi	Promote the secretion of IL‐33, thereby facilitating type 2 immune responses.	Promote	[53, 88]
LUAD	A. sydowii	Promote MDSCs, inhibit the activity of CTL and promote the accumulation of PD‐1^+^CD8^+^T cells via β‐glucan/Dectin‐1/CARD9 pathway.	Promote	[55]

Abbreviations: *A. sydowii*, *Aspergillus sydowii*; AhR, aryl hydrocarbon receptor; CRC, colorectal cancer; CTL, cytotoxic T lymphocyte; GC, gastric cancer; ILC, innate lymphoid cell; LUAD, lung adenocarcinoma; MBL, mannan‐binding lectin; MDSCs, myeloid‐derived suppressor cells; PDAC, pancreatic ductal adenocarcinoma; STAT3, signal transducer and activator of transcription; TLR, Toll‐like receptor.

### 4.1 Intratumoral Bacteria

#### 4.1.1 CRC

In CRC, previous studies have indicated that the role of bacteria in the immune microenvironment is extensive, encompassing actions on immune cells, related signaling pathways, and immune checkpoints. ITM can influence the degree of infiltration and function of immune cells. In microsatellite instability‐high (MSI‐H) CRC, the load of *F. nucleatum* DNA is inversely correlated with the density of tumor‐associated FoxP3^+^T cells and positively correlated with the ratio of M2‐like TAMs to total TAMs, but no significant correlation was found between *F. nucleatum* load and the infiltration of CD3^+^, CD4^+^, and CD8^+^T cells [59]. This suggests that *F. nucleatum* may promote tumor progression by facilitating the polarization of TAMs towards the M2 phenotype and by reducing the infiltration of related T cells. Furthermore, using the APC^min/+^ CRC mouse model, Aleksandar D. Kostic and colleagues proposed that *F. nucleatum* could recruit CD11b^+^myeloid cells to the TIME without significantly affecting the numbers of CD3^+^CD4^+^, and CD3^+^CD8^+^ lymphocytes [60]. Differentiated CD11b^+^myeloid cells, including TAMs, DCs, and tumor‐associated neutrophils (TANs), play an important role in facilitating cancer progression and angiogenesis [89], and these cells are markedly increased in the *F. nucleatum* treated mouse model [60]. Additionally, MDSCs are myeloid cells that permit tumor cell survival and are generally considered to suppress CD4^+^T cells by expressing arginase‐1 and inducible nitric oxide synthase (iNOS) to exhibit immunosuppressive activity [90, 91]. Classic myeloid DCs and CD103^+^ regulatory DCs are increased in the *F. nucleatum* ‐treated mouse model [60], which contribute to the regulation of the immune response by promoting the expansion of Foxp3^+^Treg, a CD4^+^T cell subset that can suppress cytotoxic and effector T cells, thereby inhibiting antitumor immunity [92, 93]. TAMs could inhibit T‐cell responsiveness, particularly CD4^+^T cells, through the expression of arginase‐1 [94]. Recent study also supports the role of TANs in tumor progression, angiogenesis, and the regulation of antitumor immunity [95]. Therefore, it can be inferred that *F. nucleatum* promotes the formation of a proinflammatory microenvironment conducive to CRC progression. In addition to *F. nucleatum* , a study by Amélie Lopès and colleagues indicates that the load of *E. coli* is primarily inversely correlated with the density of infiltrating CD3^+^T cells in colorectal tumors [57], which may be involved in cancer progression by inhibiting antitumor immunity.

There is a correlation between the abundance of specific ITM and the infiltration levels of T cells or suppressive immune cells. The T cells affected by the ITM can be primarily categorized into two types: CD4^+^T cells and CD8^+^T cells. CD8^+^T cells can directly kill tumor cells by recognizing specific antigens on the tumor cell surface, while CD4^+^T cells tend to influence CD8^+^T cells or secrete related factors. ITM has both positive and negative effects on CD8^+^T cells. Harmful microbes can suppress CD8^+^T cells, thereby promoting tumor growth. Conversely, probiotics can increase the infiltration of CD8^+^T cells, which contributes to immunotherapy and, to some extent, indicates a favorable prognosis. A study demonstrated that actinomyces could promote the progression of CRC by activating the TLR2/NF‐κB pathway and reducing the infiltration of CD8^+^T cells [65]. CD4^+^T cells comprise three types of cells, among which Th1CD4^+^T cells are considered to inhibit tumor development, while Th2CD4^+^T cells and Treg cells are thought to exert key roles in promoting tumor progression [10]. *F. nucleatum* has been shown to induce the release of inflammatory cytokine IL‐12 and TGF‐β in CRC, thereby increasing the number of Tregs in CRC, leading to an immunosuppressive effect [61]. Similar to T cells, the impact of TAMs on tumor progression is diverse and related to the polarization or type of M cells. Kikuchi et al. conducted a comparative analysis of multiple samples and observed a decrease in M1‐like and an increase in M2‐like TAMs in CRC tumor samples. They suspected that TAMs in the TME shift from M1‐like TAMs to M2‐like TAMs, depending on the requirements of tumor progression in CRC [61]. In their study, tumor samples from patients with enrichment of Ruminococcaceae, Faecalibacterium, Eubacterium, and Bacteroides showed an increase in M1‐like TAMs [61], which may be related to their mechanisms of action. However, as the study failed to demonstrate a significant correlation between M2‐like TAMs and these microbes, and given the previous speculation that certain microbes might promote the transformation of M1‐like TAMs to M2‐like TAMs, further research in larger cohorts is needed to explore the relationship between M1 and M2.

ITM can also trigger immunogenic cell death (ICD) to enhance immunity. ICD is a form of cell death in which dying cells release antigens and adjuvants to enhance the immune response. The key indicators of ICD include the release of ATP and nuclear high mobility group box 1 (HMBG1), exposure of calreticulin (CRT) on the cell surface, and secretion of type I IFNs [96]. These marker molecules are referred to as danger‐associated molecular patterns (DAMPs). Some studies have combined bacterial empty envelopes and the drug oxaliplatin to treat mouse models with advanced CRC. This combined strategy included a potent inhibition of tumor growth and extended survival in the mouse models by enhancing ICD [97]. Correspondingly, oncolytic virus (OVs) activity is partially related and similar to ICD. Oncolytic virus or bacteria could specifically target the TME and lyse tumor cells, release tumor antigens, DAMPs, and pathogen‐associated molecular patterns (PAMPs). They attract inherent immune cells to the sites of lesions while activating immature DCs, subsequently priming CD8^+^T cells to produce a tumor‐specific immune response [66, 67], thereby achieving tumor suppression.

Moreover, ITM can regulate immunity by participating in relevant functional pathways within TIME. In the pathways related to the ITM and the TIME, the PRR‐mediated pathways are frequently investigated. Interaction of microbial adjuvants and PRRs is involved in the regulation of the TIME. The adjuvanticity of microbes refers to the immune regulatory effects of PAMPs derived from microbes [58]. PRRs can sense PAMPs, which is a key step in the microbe‐induced innate immune response and the subsequent adaptive immune response [98, 99]. TLRs are the most studied subtype of PRRs [100]. The microbial activation of TLRs exerts a dual role in the TIME. First, ITM can drive the formation of an immunosuppressive TME through TLRs. For example, *F. nucleatum* recognized by TLR4 upregulates the IL‐6/p‐STAT3/c‐MYC signaling pathway, leading to M2‐like TAM polarization and CRC progression [62]. Second, ITM can also maintain an immunostimulatory TME through TLRs, acting as anticancer agents. TLR agonists can also, in concert with interferon γ, increase the proinflammatory cytokines TNF‐α, IL‐12p40, and IL‐12p70 while decreasing IL‐10, forming an inflammatory microenvironment to activate the antitumor immune response [101]. Of course, some chronic inflammation can be carcinogenic, such as inflammation‐induced CRC. Lactobacillus can trigger the expression of IL‐10 in a TLR6‐dependent manner to suppress colonic inflammation, thus preventing the occurrence of inflammation‐induced CRC [68]. Besides TLRs, nucleotide‐binding oligomerization domain (Nod) and Nod‐liked receptors (NLRs) are also a group of frequently studied PRRs [102]. Nod1 is a member of the NLR family and is a cytoplasmic protein expressed in various cells and functions. The activation of Nod1 promotes the proliferation of MDSCs and the expression of arginase‐1. Arginase‐1 maintains the immunosuppressive potential of MDSCs and promotes M2‐like repolarization of macrophages, ultimately forming an immunosuppressive TME [103], and promoting tumor progression.

In addition, numerous studies reveal that microbial metabolites such as short‐chain fatty acids (SCFAs), bile acids, and inosine participate in the modulation of the TIME by interacting with the receptors expressed on tumor cells and tumor‐associated immune cells. For instance, SCFAs produced by intestinal anaerobic bacteria, such as butyrate and acetic acid, can suppress protumor inflammatory responses. Specifically, butyrate can be recognized by G protein‐coupled receptors (GPR) on the surfaces of colonic cells and immune cells. In this manner, butyrate increases the levels of IL‐10 and retinoic acid in the intestinal microenvironment, promoting the differentiation of naive T cells into Treg [17]. Simultaneously, it also promotes the proliferation of Treg, thereby suppressing protumor inflammation [104]. Inosine is a purine metabolite derived from *Bifidobacterium pseudolongum* . It binds to the adenosine 2A receptor (A2AR) on T cells and initiates the inosine A2AR‐cAMP‐PKA signaling pathway. With the costimulation of DCs, inosine induces the differentiation of naive T cells into CD4^+^Th1 [69]. Compared to PD‐L1 blockade alone, the combination of inosine with a PD‐L1 blocker increases CD8^+^T cell infiltration and IFN‐γ secretion in the TIME (Table 3) [117]. It should be noted that inosine is used as an alternative energy source in effector T cells, rather than glucose. Tumor cells cannot utilize inosine, making it an ideal fuel to energize T cells to kill tumor cells [121]. Other bacterial‐derived metabolites, such as N‐acetylmuramic acid and N‐acetylglucosamine, have significant immunosuppressive effects [17]. Their roles in the TIME await further elucidation.

**TABLE 3:** Applications of ITM.

Methods		Application examples	Mechanisms	References
Add ITM	Engineered bacteria	H_2_O_2_ biosynthesizers.	Application of biological synthesis technology.	[105]
		Enhance tumor radiosensitivity.	Maintain oxygen and transform chemotherapeutic drugs activity.	[106]
		Immunotherapy.	An alternative therapeutic option. A selective vehicle for drug delivery.	[107]
	Probiotics	Modulate the intestinal environment or function.	Remodel the gut microbiota; Reduce carcinogen activating enzyme activity; Enhance the intestinal barrier function.	[107]
		Regulate TME.	*Lactobacillus reuteri* can enhance antitumor immunity, and promote ICI therapy via the I3A‐AhR‐CD8^+^CTL axis.	[108]
	FMT	Regulate TIME.	Increase the infiltration of antigen‐presenting cells.	[109, 110]
	OVs	Enhance antitumor immune capabilities.	Target tumor cells, resulting in ICD, releasing signaling molecules to recruit and activate immune cells.	[111]
Eliminate ITM	Antibiotics	Amphotericin B	Inhibit pancreatic tumor.	[112]
		MTI	Kill ITM while maintaining the balance of the patient's microbiota.	[113]
	Phage	—	Alternative therapies to antimicrobials.	[114]
		—	Bind to tumor cells or interact with fibroblasts within the TME.	[107]
Vaccine	Biomimetic nanovaccine	—	Eliminate *F. nucleatum* without affecting the intratumoral and intestinal microbiota.	[115]
Metabolic products	HBL	—	Inhibit the growth of tumors in mice.	[116]
	Inosine	—	Promoting the conversion of naive T cell to CD4^+^Th1 cell via the A2AR‐cAMP‐PKA pathway.	[69]
			Enhance the immunogenicity of tumor cells; Provide an alternative carbon source for CD8^+^T cells.	[117]
	SCFAs	Butyric acid	Recognized by GRP, promotes the differentiation and proliferation of Treg cells.	[17, 104]
			Increase the IFN‐γ and granzyme B in CD8^+^T cells; Improve the efficiency of ICI.	[118]
			Inhibit HDACs.	[119]
	Tryptophan metabolism	Indole	Activate the AhR activity.	[79, 108]
		I3A	Promote IFN production; Enhance ICI therapy.	[79, 108]
		3‐ICA	Boost anti‐PD1 efficacy.	[120]
Bacterial‐fungal interactions	Candida and Lactobacillus	—	Affect the pathogenicity.	[51]
	Yeast and *H. pylori*	—	May provide a potential therapeutic approach.	[51]

Abbreviations: 3‐ICA, indole‐3‐carboxylic acid; AhR, aryl hydrocarbon receptor; CRC, colorectal cancer; CTL, cytotoxic T lymphocytes; FMT, fecal microbiota transplantation; *H. pylori, Helicobacter pylori;* HBL, hemolysin BL; HDACs, histone deacetylases; I3A, Indole‐3‐aldehyde; ICD, immunogenic cell death; ICI, immune checkpoint inhibitor; ITM, intratumoral microbiota; MTI, metronidazole‐triazole‐iodoacetamide; Ovs, oncolytic virus; SCFAs, short‐chain fatty acids; TIME, tumor immune microenvironment; TME, tumor microenvironment.

Additionally, ITM can enhance or suppress immune function by influencing immune checkpoints. Immune checkpoints exert their effects by deactivating immune cells. Inhibitory checkpoints mainly include PD‐1, PD‐L1, CTLA‐4, TIM‐3, LAG‐3, TIGIT, CEACAM1 (carcinoembryonic antigen‐related cell adhesion molecule 1) and CD47. Several studies found that *F. nucleatum* could inhibit the activity of NK cells and cytotoxic T cells through the interaction between Fap2 and TIGIT, or between Fap2 and CEACAM1 [63, 64]. Fap2 is a protein originating from *F. nucleatum* [63]. In addition to *F. nucleatum* , other bacteria, such as pathogenic Neisseria, can also bind to CEACAM1 [17]. Another checkpoint, CD47, is expressed on the surface of tumor cells. SIRPα, the ligand for CD47, is expressed on DCs and macrophages. The CD47‐SIRPα interaction can inhibit antigen presentation and phagocytosis. Intravenous injection of Bifidobacterium into tumor‐bearing mice can upregulate the expression of IFN‐I in DCs via the stimulator of interferon genes (STING). IFN‐I is a crucial cytokine for antigen cross‐presentation and T‐cell activation. In vivo injection of antibiotics that clear Bifidobacterium can reduce the efficacy of CD47 blockade, suggesting that Bifidobacterium may serve as a potential adjuvant for CD47 blockade [47]. In summary, in CRC, the ITM not only encompasses a wide variety of species with significant abundance but also exhibits diverse mechanisms of action, all of which are closely associated with the TIME. A thorough understanding of their interaction mechanisms will provide new insights and strategies for the diagnosis, treatment, and prognosis of cancer.

#### 4.1.2 Melanoma

Melanoma is extensively studied for its association with various ITM. In melanoma‐related studies, it has been found that ITM plays a role in antigen immune responses within the TIME. The antigen immune response is one of the most common forms of immune response. Successful induction of a related adaptive antitumor response requires two key factors: Human leukocyte antigens (HLA) and activated T cells. The process begins with HLA presenting antigens to activate CD8^+^T cells, which then recognize and kill tumor cells presenting the antigens. Notably, bacterial peptides are considered exogenous compared to tumor cell antigens, which are more likely to elicit an immune response [122]. Kalaora et al. identified microbes in melanoma through sequencing, discovering that ITM produced bacterial peptides that could be presented by HLA‐I and HLA‐II, subsequently activating CD4^+^ and CD8^+^T cell activity [70]. The discovery of these peptides to some extent suggests that microbes can alter tumor surface antigens. This study also found that HLA‐I could present 11 bacterial peptide antigens from *F. nucleatum* , *Staphylococcus aureus* , and *Staphylococcus epidermidis* , which show similar patterns in both primary and metastatic tumor lesions from different patients [70]. Moreover, there is not only similarity among bacterial peptide antigens, but microbial antigens also share similar epitopes with tumor antigens. Antigen mimicry is a phenomenon where microbes express these shared epitopes, a process known as cross‐reactivity [98]. Alexandra Snyder and colleagues analyzed neoantigen epitopes in melanoma patients with different prognosis, finding that some neoantigen epitopes are homologous to microbial epitopes, with higher homology correlating with better clinical outcomes [123]. Another study revealed that *Bifidobacterium breve* , with the epitope SVY, could induce the generation of SVY‐specific T cells, which recognize and attack melanoma cells expressing the epitope SIY. Elimination of *Bifidobacterium breve* promoted the growth of melanoma cells [71]. These findings suggest that antigen mimics exist within tumors and may influence antitumor immune responses. In addition, in melanoma, ITM is also associated with the infiltration of immune cells. Research by Gongjian Zhu et al. has shown that the presence of various bacteria in skin melanoma was associated with the activity of CD8^+^T cells. The ITM could modulate chemokine levels and affect CD8^+^T cell infiltration, thereby influencing the progression of skin melanoma. The intratumoral bacterium Lachnoclostridium was ranked first in positive correlation with infiltrating CD8^+^T cells, followed by Gelidibacter, Flammeovirga, Acinetobacter, and Tropobacter. These ITM were closely related to the expression of chemokines including CXCL9, CXCL10, and CCL5 [72]. High abundance of Lachnoclostridium significantly reduced the risk of mortality, with no statistically significant correlation observed between its abundance and levels of NK cells, B cells, or CD4^+^T cells, indicating the influence primarily through CD8^+^T cells. Additionally, they noted a negative correlation between Algibacter and Epilithonimonas and CD8^+^T cell engagement [72].

#### 4.1.3 Lung Cancer

Lung cancer is one of the leading causes of cancer‐related mortality, and the lungs, as the largest mucosal tissue exposed to the external environment, are subject to various airborne microbes and environmental damage. Therefore, the relationship between the host's response to these microbes and lung tumorigenesis merits attention. ITM can influence tumor cells through antigenic immune responses. Shin‐Heng Chiou and colleagues analyzed T‐cell receptor (TCR) sequencing data from 178 lung cancer patients. They found that some proteins that cross‐react with EBV and *E. coli* were over‐expressed in tumor tissues compared to normal tissues, and their findings suggest that this cross‐reactivity may underlie the presence of virus‐specific T cells in tumor infiltrates and that pathogen cross‐reactivity may be a feature of multiple cancers [73]. Additionally, ITM can affect immune cells through metabolic pathways. Chengcheng Jin et al. established a germ‐free mouse model of lung cancer, revealing that commensal bacteria could stimulate myeloid cells to produce IL‐1β and IL‐23, inducing the proliferation and activation of Vγ6^+^Vδ1^+^γδT cells, which in turn produce IL‐17 and other effector molecules, promoting inflammation and tumor cell proliferation. Notably, common bacterial genera such as Staphylococcus, Streptococcus, Lactobacillus, Pasteurellaceae, were found to be significantly enriched in tumor‐bearing lungs, potentially contributing to tumor progression [74]. Shuo Shi et al. discovered positive correlations between Lachnoclostridium, Acinetobacter, and Paenibacillus with M1 macrophages, while Aeromonas and Vibrio showed negative correlations with Treg [75]. Furthermore, in a lung cancer mouse model, Yuting Deng et al. reported that TLR1/TLR2 expression predicts favorable prognosis in lung cancer patients, and demonstrated that bacterial lipoproteins (BLP) could activate TLR2, resulting in the reprogramming of MDSCs to differentiate into inflammatory M1 phenotypes through JNK pathway [124], thereby blocking their immunosuppressive functions and inhibiting tumor progression.

#### 4.1.4 Gc

In the past, *H. pylori* was predominantly regarded as the main bacterium associated with the stomach. However, with advancements in technology, other microbes in GC have come into focus, revealing their roles through various studies. One study showed that the abundance of genera Stenotrophomonas and Selenomonas in the gastric mucosa displayed a positive correlation with the density of tumor‐associated BDCA2^+^ plasmacytoid dendritic cells (pDCs) and Foxp3^+^Tregs [76], thereby promoting tumor development. Similar to the role of Fap2 and TIGIT in CRC, Chamutal Gur et al. found that *H. pylori* can interact with CEACAM1 through its outer membrane protein HopQ, leading to inhibition of immune cell [77]. CEACAMs are inhibitory receptors predominantly expressed by activated T cells and NK cells. The interaction between HopQ and CEACAM1 facilitates the translocation of the virulence factor cytotoxin‐associated gene A (CagA) into host cells. CagA activates NF‐κB‐dependent inflammatory signaling pathways, enhancing the release of proinflammatory mediators such as IL‐8 and recruiting inflammatory cells to the gastric mucosa, which contributes to chronic inflammation. Moreover, CagA could also activate a mitogenic signaling pathway including PI3K‐AKT, SHP2, and WNT‐β‐catenin signaling to promote cell proliferation [125]. Through this process, *H. pylori* can play a significant role in facilitating tumorigenesis. Collectively, these microbes serve as risk factors for GC development.

#### 4.1.5 PDAC

The pancreas is one of the organs where microbes congregate, including genera such as Pseudomonas, Curvibacter, Streptococcus, Sphingomonas, and Corynebacterium. In PDAC tissues, the phyla Proteobacteria and Firmicutes are shown to be highly enriched [126]. In PDAC, ITM can also affect the infiltration and function of immune cells in TIME through antigen immune responses or signaling pathways. Accumulating evidence suggests that the ITM can promote the immunosuppressive features of PDAC. Shohei Abe et al. observed a correlation between the presence of three anaerobic bacteria—Bacteroides, Lactobacillus, and Peptoniphilus—with tumor immune suppression and poor prognosis in PDAC [127]. ITM could reduce CD8^+^T cell levels and lower immune reactivity in the TME via the IFN pathway, thereby facilitating tumor growth [58, 78]. In mouse models of pancreatic cancer, ITM selectively activated TLRs in monocytes to induce M2‐like TAM differentiation. Antibiotic clearance of ITM was associated with immunogenic reprogramming of the PDAC TME, which could promote TH1 differentiation of CD4^+^T cells and CD8^+^T cell activation, M1‐like TAM differentiation, and reduce intratumoral MDSCs [10], thus inhibiting tumor progression. Erick Riquelme et al. found that the ITM shapes immune responses, thereby promoting T cell activation. Utilizing immunohistochemistry and immunofluorescence staining techniques, they delineated the tumor immune infiltrates. Subsequent statistical analysis revealed a significant positive correlation between the tissue densities of CD8^+^T cell, and GzmB^+^ cells and overall survival in patients with PDAC, and they observed a strong significant correlation between the tissue densities of CD8^+^T cell and GzmB^+^ cells with ITM diversity. The study tested ITM, including the genera Saccharopolyspora, Pseudoxanthomonas, and Streptomyces, which were found to be positively associated with the density of CD8^+^T cell [128]. In addition, studies have also shown that microbes can influence the progression of PDAC through metabolism. Tumor metabolism is a crucial factor in the growth and development of tumors. And it has been demonstrated that microbe imbalances can induce systemic metabolic alterations [129]. Hezaveh and colleagues investigated the impact of the aryl hydrocarbon receptor (AhR) on the function of TAMs in PDAC. AhR acts as a sensor for tryptophan metabolites produced by the microbe. The absence of AhR in myeloid cells or the pharmacological inhibition of AhR diminished PDAC growth, augmented the therapeutic efficacy of immune checkpoint blockade, and increased the intratumoral frequency of IFNγ^+^CD8^+^T cells. Hezaveh et al. demonstrated that AhR is a key driver of the TAM protumor phenotype, and when AhR function is abolished, TAM polarization shifts to a proinflammatory state. By comparing different mouse models, Hezaveh et al. found that tryptophan‐producing bacteria, such as Lactobacilli, increased AhR transcriptional responses in PDAC, promoting an immunosuppressive TME and tumor progression [79].

#### 4.1.6 Others

In addition to the tumors mentioned above, the presence of ITM has also been reported in other tumors. In bladder cancer, clearance of microbes could lead to the increased infiltration of Treg, induce immune suppression, and promote tumor progression [80]. TLR4 recognition of bacterial lipopolysaccharides could induce hepatocyte expression of CXCL1, a chemokine that recruits CXCR2^+^ polymorphonuclear MDSCs, thereby creating an immunosuppressive environment to promote the development of cholangiocarcinoma (CCA) in a murine model [81]. TLR5 sensing of bacterial flagellin can trigger inflammation and skin cancer by upregulating a protein known as high mobility group box 1 [82]. Research by Qian Zhang et al. identified that Proteobacteria, Actinobacteria, Firmicutes, and Bacteroidetes are the most abundant phyla in nasopharyngeal carcinoma (NPC) tissues, with certain microbes exhibiting heightened sensitivity to systemic inflammatory and immune state changes. For example, Shewanella, Rhodobacter, and Altererythrobacter showed higher abundance in high neutrophil infiltration groups, suggesting a potential correlation [83]. Lauren M. Perry et al. conducted research on soft tissue sarcomas (STS). Despite limited detailed characterization of the ITM in STS, they identified the presence of bacterial phyla such as Proteobacteria, Bacteroidetes, and Firmicutes, as well as various viral species within the tumors. A significant positive correlation was observed between the relative abundance of viruses, such as HHV‐6 and respirovirus, with the infiltration of NK cells in the TME [84]. Additionally, the *Mycoplasma penetrans* HF‐2 permease has high homology with tumor antigen MA6, capable of stimulating CD4^+^T cells to attack MA6‐expressing tumor cells [130]. Aurélie Fluckiger and colleagues detected T cells in cancer patients that can cross‐recognize tumor antigens and microbial antigens. They also identified a phage antigen called the ribbon protein, which shares similar epitopes with the tumor‐associated antigen PSMB4. In mouse models, MHC‐I presented the ribbon protein to induce a specific CD8^+^T cell response, which then recognizes and attacks PSMB4‐expressing tumor cells, enhancing PD‐1 blockade efficacy, and prolonging mouse survival [131].

### 4.2 Fungi

The fungal community can modulate the host immune response, which is significant in inflammatory diseases and tumor progression (Table 2). Immune cells, including DCs, macrophages, and NK cells, can recognize the carbohydrate components of fungal cell walls, such as β‐glucans or mannan, through their PRRs, forming the first line of defense against fungal infections. To explore the potential presence of fungal interaction networks and coabundant taxa, Anders B. Dohlman et al. applied SparCC and found that *C. albicans* and *S. cerevisiae* were each at the center of two anticorrelated coabundance clusters, which span all GI cancer types. The coabundance group associated with *C. albicans* included C. dubliniensis, *C. tropicalis* , and *C. guilliermondii* , while the group associated with *S. cerevisiae* was comprised of taxa including S. eubayanus, C. jadinii, P. membranifaciens, as well as *C. parapsilosis* and *C. glabrata* . They also found that these two coabundance clusters were predictive of host gene expression across head and neck, stomach, and colon cancers [51]. These findings suggested that cancers of the GI tract may segregate into Candida and Saccharomyces associated tumors. Classifying GI tumors into Ca‐type and Sa‐type for further analysis, Anders B. Dohlman et al. found that in GC, genes associated with cytokine interactions, host immunity, and inflammation were enriched in Ca‐type tumors, including IL1A, IL1B, IL6, IL8, CXCL1, CXCL2, and IL17C [51]. And this pro‐inflammatory immune profile is consistent with the previous proposal by Li et al. that *Candida albicans* calls for IL‐1β, neutrophil and Th17 cell infiltration in the gut [132]. Additionally, in stomach cancers, Anders B. Dohlman et al. found that Candida was associated with the expression of genes involved in cytosolic DNA sensing, TLR signaling, and Nod‐like receptor signaling. In CRC, they found that tumor suppressor genes and genes regulating the cell adhesion pathway were downregulated in Ca‐type tumors, including PTK2B, CDKN2C, and NET1, while genes such as BMP15, PFN3, CCL27, PIP, and SAGE1 were upregulated in Ca‐type tumors [51]. These suggest a role for fungi in the pathogenesis of GI cancers. In addition, Anders B. Dohlman et al. found that in head and neck cancers, the tumor suppressors TP53 and CDKN2A as well as fibronectin were expressed at a lower level in Ca‐type tumors, while they also saw upregulation of IL22, IL24, CARD10, and CD44 expression in Ca‐type tumors, whereas there was no upregulation in Sa‐type tumors. Suggesting that the presence of Candida in head and neck cancers is associated with reduced expression of genes related to cell adhesion molecules [51].

**FIGURE 3:**
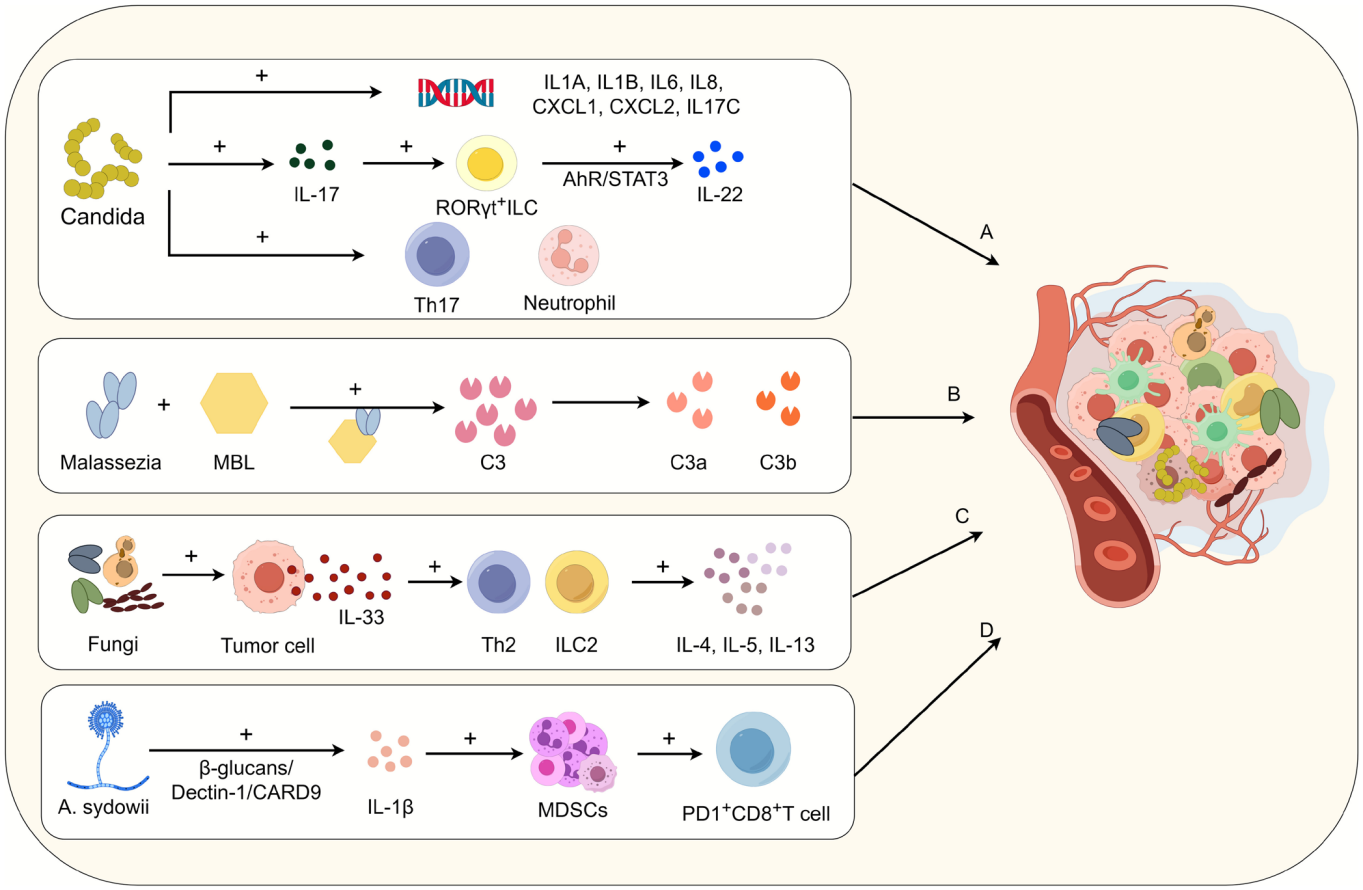
Fungi can affect tumor progression by modulating the tumor immune microenvironment (TIME). (A) In Candida‐type tumors, genes associated with cytokine interactions, host immunity, and inflammation—such as IL1A, IL1B, IL6, IL8, CXCL1, CXCL2, and IL17C—are enriched and involved in host immune regulation; Candida can trigger glycolysis in macrophages and induce the secretion of IL‐7, thereby promoting IL‐22 production by RORγt^+^ILCs via the AhR and STAT3 pathways, which facilitates tumor formation. Additionally, Candida induces the infiltration of IL‐1β, neutrophils, and Th17 cells in the gut, contributing to tumor progression. (B) Fungal pathogens such as Malassezia can adhere to MBL, activate the complement cascade, and cleave C3 into C3a and C3b, thereby promoting tumor progression. (C) Intratumoral fungi can stimulate tumor cells to secrete IL‐33, which promotes the infiltration of Th2 cells and ILC2s and further drives tumor growth. (D) A. sydowii promotes the expansion and activation of MDSCs through IL‐1β mediated by the β‐glucan/Dectin‐1/CARD9 pathway, thereby facilitating tumor progression. AhR, aryl hydrocarbon receptor; CTL, cytotoxic T lymphocyte; ILCs, innate lymphoid cells; MBL, mannan‐binding lectin; MDSCs, myeloid‐derived suppressor cells; TIME, tumor immune microenvironment.

*Candida albicans* can play a carcinogenic role in Dectin‐3 deficient mice. Its elevated fungal load triggers glycolysis in macrophages and IL‐7 secretion. IL‐7 induced IL‐22 production in RORγt^+^ innate lymphoid cells (ILCs) via AhR and the signal transducer and activator of transcription (STAT3) pathway, eventually modulating host immunity and facilitating colon tumorigenesis [85]. Aykut et al. elucidated the role of fungi by utilizing 18S rRNA internal transcribed spacer sequencing, and demonstrated that both murine and human tumors exhibit higher fungal abundance and distinct fungal compositions compared to normal pancreatic tissue, along with GI translocations. Furthermore, the study found that oral administration of amphotericin B in spontaneous or orthotopic pancreatic cancer mouse models correlated with delayed tumor onset and growth, and enhanced the chemotherapy efficacy of gemcitabine. Mechanistically, the pathogens of fungi could adhere to mannan‐binding lectin (MBL) to activate the complement cascade and cleave C3 into C3a and C3b, thereby promoting carcinogenesis and tumor progression [86, 87]. In addition to targeting the complement system, Aftab Alam et al. identified the proinflammatory cytokine IL‐33, which is released as a chemotactic agent for Type 2 immune cells in response to intratumoral fungi. Intratumoral fungi could stimulate PDAC cells to secrete IL‐33. Alam et al. discovered that IL‐33 is a downstream target of KrasG12D. The secretion of IL‐33 could promote a Type 2 immune response, and accelerate progression of PDAC [53]. TH2 cells and ILC2 can further promote tumor growth by secreting tumor‐promoting cytokines such as IL‐4, IL‐5, and IL‐13. In contrast, deletion of IL‐33 gene or antifungal treatment contributed to the decrease of TH2 and ILC2 infiltration, leading to inhibition of tumor development and prolongation of the survival in PDAC patients [53, 88]. This mechanism suggested that the IL‐33‐ILC2/TH2 axis might provide a promising therapeutic target for PDAC. But notably, although it has been shown that intratumoral fungi promote IL‐33 secretion, it remains unclear whether live fungi are required or if fungal components are sufficient to drive this mechanism [53, 88]. Ning‐Ning Liu et al. identified the enriched tumor‐resident Aspergillus sydowii (A. sydowii) in lung adenocarcinoma (LUAD) patients using three different isogenic lung cancer mouse models; they found that A. sydowii promoted lung tumor progression via IL‐1β‐mediated amplification and activation of MDSCs, thereby inhibiting the activity of cytotoxic T lymphocytes (CTL) and promoting the accumulation of PD‐1^+^CD8^+^T cells. This process was mediated through the β‐glucan/Dectin‐1/CARD9 pathway. Blocking IL‐1β can reverse A. sydowii‐induced immunosuppression. Analysis of human samples confirmed that the presence of A. sydowii was associated with immune suppression and poor prognosis of the patients [55]. In conclusion, although fungi have a lower biomass in tumors than bacteria and many of the fungal‐related mechanisms identified so far require further substantiation, the role of fungi in tumorigenesis and development cannot be ignored (Figure 3).

## 5 Impacts of ITM on Tumor Therapy

### 5.1 Immunotherapy

Immunotherapy is a treatment modality that targets the human immune system, aiming to modulate and activate the body's immune response. It primarily consists of four major categories: immune checkpoint inhibitors (ICIs), tumor vaccines, cellular immune cell therapy, and nonspecific immunomodulators [107]. The impact of the intratumoral microbiota (ITM) on ICIs has been a subject of significant interest. The ITM can affect the therapeutic outcomes of immunotherapy by altering the local immune environment within the tumor tissue [2]. Observations of the efficacy of immunotherapy in melanoma, bladder cancer, renal cancer, and lung cancer have sparked interest in how the microbiota can enhance the efficacy of immunotherapy. The origins of immunotherapy stem from an understanding of the coadaptation between the host and microbes, utilizing bacteria to trigger the immune system to attack and destroy cancer [133]. Prior research indicates that the local microbiota can induce inflammation associated with LUAD by activating lung‐resident T cells. Lactobacillus activates NK T cells to promote cellular immunity in breast cancer [134]. The microbiota from responders to immune checkpoint blockade can shape the landscape of mononuclear phagocytic cells within the tumor microenvironment (TME) [135]. Jiang et al. recently demonstrated that succinic acid produced by *F. nucleatum* reduces the sensitivity of CRC patients to PD‐1 inhibitor therapy by affecting CD8^+^T cell‐mediated antitumor immunity [136].

### 5.2 Chemotherapy

In the current treatment of advanced and terminal cancer, chemotherapy is the primary modality of therapy. Chemotherapy resistance is a significant cause of mortality in many cancer patients. A substantial body of research has identified a correlation between the ITM and chemotherapy resistance, suggesting it as an attractive target for enhancing the efficacy of chemotherapy [126]. The main mechanisms by which ITM contribute to chemotherapy resistance include: (1) Metabolism: ITM affect the active form of chemotherapeutic agents by regulating various specific enzymes [107]. (2) Immunity and Inflammation: Microbes can activate immune cells and influence the efficacy of chemotherapeutic drugs through inflammatory responses and antitumor immune regulation [107]. (3) Autophagy: Iida et al. conducted a comparative study on patients with CRC who had relapsed after chemotherapy and those who had not, and found that the number of *F. nucleatum* in relapsed patients increased. The study pointed out that *F. nucleatum* affects CRC through the TLR4 and myeloid differentiation primary response 88 (MYD88) pathways. This, in turn, activates autophagy, thereby promoting chemotherapy resistance in CRC patients [107]. Aykut et al. found that fungal ablation can enhance the therapeutic efficacy of gemcitabine chemotherapy [87]. In summary, the diversity of ITM exerts both positive and negative effects on chemotherapy. The application of precise and personalized microbiota‐targeted adjuvant treatments may potentially reverse chemotherapy resistance and enhance the efficacy of chemotherapy.

### 5.3 Radiotherapy

Radiotherapy for cancer utilizes ionizing radiation to exert cytotoxic effects on tumor cells, aiming to reduce tumor size and achieve other therapeutic objectives [137]. Similar to chemotherapy, radiotherapy is a crucial adjuvant treatment for advanced and terminal solid tumors. In certain cancers, such as nasopharyngeal cancer, radiotherapy can also be the treatment of choice. In 2019, Uribe‐Herranz et al. discovered that vancomycin can enhance the antitumor immune response induced by radiotherapy and inhibit tumor growth [107]. In 2021, Shiao et al. used a combination of antibiotics to eliminate commensal bacteria which led to the growth and expansion of commensal fungi and reduced the efficacy of radiotherapy [138]. In the same year, Dong et al. used metronidazole to eliminate *F. nucleatum* , significantly improving the effectiveness of radiotherapy [139]. Some studies suggest that dietary therapy, antibiotic application, fecal microbiota transplantation (FMT), and oral microbiota transplantation (OMT) are promising adjuvant methods to improve radiotherapy outcomes [107]. Consequently, based on improving radiotherapy outcomes while reducing surrounding tissue damage, further research and exploration are needed to individually select beneficial microbiota.

## 6 Applications of ITM

### 6.1 Engineered Bacteria

Engineered bacteria serve multiple functions (Table 3). They can be employed in monotherapies such as producing specific enzymes, cytokines, or other molecules to modulate the tumor microenvironment (TME), enhance immune responses, or directly kill cancer cells. They can also increase the sensitivity of certain tumor cells to chemotherapy or radiotherapy, or act as drug delivery vehicles to target tumor cells for treatment. Bacteria possess the capability of self‐replication, and when combined with synthetic biology techniques, they can be developed into H_2_O_2_ biosynthesizers for cancer treatment [105]. Furthermore, they can enhance tumor radiosensitivity by maintaining oxygen supply and converting inactive chemotherapeutic drugs into active forms [106]. Conventional treatments face substantial challenges due to harmful deep tissue penetration and primary or acquired resistance. Targeted engineered bacteria for tumor treatment can offer an alternative therapeutic option. Beyond the aforementioned, engineered bacteria can also serve as a conceivable therapeutic probe and as a selective vehicle for drug delivery [107]. A bioengineered strain of *Escherichia coli* ( *E. coli* ) can effectively increase the concentration of L arginine in the TME and enhance T‐cell infiltration, thereby improving the efficacy of immunotherapy [140]. Furthermore, nonpathogenic *E. coli* can be engineered to specifically lyse and release anti‐CD47 nanobodies within the TME. This strategy targets CD47‐mediated tumor immune evasion, boosts the activity of tumor‐associated T cells, promotes tumor regression, and helps prevent metastasis [141]. An engineered attenuated *Salmonella typhimurium* strain that secretes *Vibrio vulnificus* flagellin B within tumor tissue effectively inhibited tumor growth and metastasis in mouse models, significantly prolonging survival. This effect is associated with the TLR4 signaling pathway, promoting the transition of macrophages from the M2 to the M1 phenotype [142]. Engineered *Lactococcus lactis* can secrete the Flt3L‐OX40L fusion protein, which promotes dendritic cell (DC) infiltration and activates CD8^+^T cells within the tumor immune microenvironment (TIME). This not only converts immunologically “cold” tumors into “hot” tumors but also synergizes with anti‐PD‐1 therapy to enhance treatment efficacy [143]. However, despite the powerful therapeutic effects demonstrated by engineered ITM, the underlying mechanisms remain incompletely understood, and most studies are still in the preclinical stages. Determining the optimal dosage and administration frequency continues to pose challenges. In the future, conducting more human‐based studies will help establish safe dosing regimens and enable precise control over these bacteria.

### 6.2 Probiotics

Certain probiotics, such as Lactobacilli, have been found to play a significant role in cancer prevention (Table 3) [144]. Other microbes, including Bifidobacteria and yeasts, also contribute to cancer prevention [145]. The mechanisms of action of probiotics primarily include: (1) remodeling of the gut microbiota; (2) regulation of intestinal conditions; (3) enhancement of the intestinal barrier function; (4) enhancement of host immunity; (5) regulation of signaling pathways; (6) modulation of the metabolic status of the TME; (7) regulation of tumor cell apoptosis [107]. Notably, recent reports indicate that a probiotic strain named *Lactobacillus reuteri* , according to a method described by Bender and colleagues, can enter the TME of solid tumors from the small intestine, enhance antitumor immunity, and promote ICI therapy through its metabolite indole‐3‐aldehyde (I3A) via the I3A‐AhR‐CD8^+^CTL axis [108]. Simultaneously, Zhang et al. have demonstrated that a probiotic strain *Lactobacillus plantarum* L168 and its metabolite indole‐3‐lactate can accelerate the production of IL12a in DCs, alter chromatin accessibility, thereby enhancing the function of tumor‐associated CD8^+^T cells [146]. The mechanisms of action of probiotics and synthetic bacteria have been extensively studied in recent years. Probiotic‐mediated chimeric antigen receptor T‐cell (CAR‐T) therapy is an emerging treatment strategy that combines the biological regulatory properties of probiotics with the targeted killing function of CAR‐T cells, bringing new hope for cancer treatment. Vincent et al. designed a CAR target synthesized by *Escherichia coli* Nissle (EcN), which has been proven to be safe and effective in multiple human and mouse cancer models. This system is delivered to the tumor site by bacteria and released in situ [147]. This target could broadly bind to cell surface and matrix proteins of TME, and the CAR‐T cells were able to recognize targets released by EcN, thereby activating CAR‐T cells and triggering a strong antitumor immune response. A clinical study also reported that Bifidobacterium appears to improve clinical outcomes in patients with metastatic renal cell carcinoma receiving immunotherapy. However, larger scale studies are still needed to confirm this observation and elucidate the underlying mechanisms [148]. As research on probiotics continues to expand, they may play an increasingly critical role in the future, particularly in anticancer applications. Nevertheless, debates regarding the efficacy of probiotics persist, and this article also mentions the varying effects of Lactobacillus on tumors. These conflicting results may be attributed to strain‐specific characteristics of the commensal microbiota. Future breakthroughs may be achieved through personalized probiotic formulations. Moreover, integrating nanotechnology and genetically engineering probiotics as targeted delivery vehicles could further advance the development of probiotics.

### 6.3 FMT

Fecal microbiota transplantation (FMT) has been demonstrated to have both preventive and therapeutic effects on cancer (Table 3). Rosshart and colleagues transplanted the intestinal microbes from wild mice into experimental mice, resulting in enhanced resistance to inflammatory CRC in the recipient mice [149]. Riquelme and others found that the survival time of patients with PDAC was associated with the composition and diversity of the intratumoral microbiota (ITM) [128]. The primary mode of action by which FMT affects immunotherapy is through the regulation of the tumor immune microenvironment (TIME), including both innate and adaptive immunity. FMT has been found to increase the infiltration of antigen‐presenting cells into the TME, leading to the release of chemokines and the activation of T cells. Furthermore, research shows that FMT can overcome the resistance of melanoma patients to anti‐PD‐1 therapy, which may be achieved by enhancing the activation of CD8^+^T cell and reducing the frequency of myeloid cells expressing IL‐8 in the TME [109, 110]. Besides reprogramming the TIME, the therapeutic effects of FMT are also associated with microbial metabolites [107]. In recent years, FMT has been widely used in the treatment of refractory colitis, including immune‐related colitis. Its immunomodulatory effects have gained increasing recognition and have been explored as a potential therapeutic approach for metastatic malignancies. Furthermore, clinical trials have indicated the role of FMT in melanoma, with some advanced melanoma patients overcoming resistance to anti‐PD‐1 therapy after undergoing FMT [109, 150]. However, the mechanisms of action of FMT, ethical issues, and treatment protocols remain unclear, and more high‐quality randomized controlled trials and clinical studies are needed to clarify these controversies.

### 6.4 OVs

Oncolytic virus (OVs) are classified as naturally occurring or genetically modified viruses that replicate within cancer cells and kill them without affecting healthy cells (Table 3). OVs function by selectively replicating within tumor cells, delivering various eukaryotic transgenic payloads, inducing immunogenic cell death (ICD), and enhancing anticancer immunity [151]. In detail, OVs target selective tumor cells, resulting in ICD, releasing progeny viral particles, re‐infecting adjacent tumor cells, viral and tumor‐associated antigens, DAMPs, cytokines, and interferons, thereby recruiting and activating DCs, NK cells, and T cells, enhancing antitumor immune capabilities [111]. Currently, four OVs and one nononcolytic virus have been authorized to treat cancer in different parts of the world. They are talimogene laherparepvec (T‐VEC), H101, ECHO‐7 (Echovirus), Teserpaturev, and one nononcolytic virus (Nadofaragene firadenovec) [151]. In recent years, ongoing clinical trials involving OVs, including herpes simplex virus (HSV) and vaccinia virus, have demonstrated their potential in treating various malignant tumors [152]. However, translating OVs therapy from preclinical research to clinical application faces challenges in terms of safety and efficacy. Ensuring the safety of OVs is critical, as uncontrolled viral replication and off‐target infections may lead to adverse toxicities. To address these safety concerns, it is essential to engineer OVs for tumor‐specific targeting through genetic modifications, such as incorporating tumor‐specific promoters or deleting genes essential for replication in normal cells [152]. Host immune responses, tumor heterogeneity, and administration methods are all factors that influence therapeutic efficacy. The development of novel delivery systems, such as nanoparticles or cellular carriers, as well as combining OVs with existing immunotherapies, holds promise for improving targeting and distribution. Additionally, optimizing dosing, enhancing viral tropism, and utilizing ideal preclinical models—such as patient‐derived xenograft models—can facilitate the translation from preclinical studies to clinical applications. In summary, OV therapy is widely regarded as a promising emerging cancer treatment method. Despite its tumor specificity, nonpathogenicity, unique pharmacokinetics, strong operability, and low potential for drug resistance, challenges such as the risk of virus leakage and unintentional spread, strict transportation and storage conditions, and special OVs injection methods need to be overcome [151]. Therefore, further experimentation and research are required to elucidate the mechanisms and address these issues.

### 6.5 Antibiotics

The impact of the ITM on tumor progression has been extensively studied, and correspondingly, antibiotic therapy targeting the ITM is an investigative treatment approach (Table 3). For example, the antifungal drugs amphotericin B, administered orally and by inhalation, effectively inhibit the growth of pancreatic tumors [112]. Since the tumor interior is hypoxic and the ITM is mainly anaerobic, metronidazole‐triazole‐iodoacetamide (MTI) is undoubtedly one of the best candidates for controlling the ITM, which could be linked to other molecules sensitive to other tumors. However, while the administration of antibiotics suppresses the growth of tumor‐associated bacteria, the systemic distribution of antibiotics can lead to an imbalance of other microbes in the body, resulting in the development of new diseases. Due to poor selectivity and potential adverse reactions, the direct use of antibiotics and other in vivo antimicrobial strategies to enhance cancer treatment often leads to uncertain outcomes. Therefore, some studies have focused on the administration of antibiotics and other drugs. Metronidazole, an antibiotic against a wide range of anaerobic bacteria, was combined with fluoropyridine to form the amphiphilic small molecule metronidazole–fluoropyridine, which self‐assembles into metronidazole–fluoropyridine nanoparticles (MTI‐FDU) in aqueous solutions. The high levels of GSH in the TME cause the disulfide bonds in the linker to break, thereby releasing the antibiotic primarily in the TME and exerting its effect. Chunxiao Gao designed and synthesized MTI‐FDU nanoparticles, achieving a synergistic antitumor effect through dual targeting of the ITM and tumor cells, and demonstrated good tumor suppression and intratumoral bacterial removal capabilities in the mouse models without causing an imbalance in the gut microbiota [113]. This indicates that antibiotic‐containing nanoparticles have clinical advantages in treating bacterially infiltrated tumors, effectively killing ITM while maintaining the balance of the patient's microbiota. In addition, clinical trials are underway to investigate the efficacy and safety of combining antibiotics with immune checkpoint inhibitors (ICIs) [153, 154, 155]. Furthermore, as mentioned earlier, the ITM regulates tumor immune responses in both positive and negative ways. The indiscriminate use of antibiotics to eliminate the microbiota may disrupt microbial balance and lead to unintended adverse outcomes, potentially promoting drug resistance or accelerating tumor progression. Therefore, the decision to use antibiotics must be grounded in a comprehensive understanding of how ITM modulates tumor immunity, guiding whether to enhance or eliminate specific microbial populations in clinical practice. Moreover, as noted above, approaches such as conjugating antibiotics with small molecules into nanoparticles to enhance antibiotic targeting or modifying delivery methods and adding targeting molecules to reduce systemic distribution can help minimize off site microbial imbalance. These strategies are expected to facilitate the future application of antibiotics.

### 6.6 Phage

Phage are a type of virus that parasitizes microbes such as bacteria, fungi, archaea, and spirochetes. Due to specific bacterial infections, phages have received considerable attention in recent years as an alternative to antimicrobial therapy, particularly in cases of antibiotic resistance (Table 3). Additionally, phages are suitable for the development of novel nanostructures and biomaterials, promoting advancements in biomedicine, such as molecular targeting, cancer diagnosis and treatment, drug and gene delivery [114]. Previous studies have found that phages can accumulate in tumor tissue to inhibit tumor growth. Subsequently, they were found to be capable of binding to tumor cells or interacting with fibroblasts within the TME [107]. Studies have demonstrated that modified M13 filamentous bacteriophages can serve as receptors in place of antibodies, enabling early cancer detection directly in bodily fluids. Additionally, phages can be utilized in the development of preventive and therapeutic vaccines, such as HER2 displaying M13 bacteriophages targeting breast cancer [107]. Similar to antibiotics and OVs, special attention must be paid to issues regarding the safety, dosage, and target populations when applying phages. Phage therapy has considerable potential development in the field of cancer treatment, which requires further validation and determination of specific mechanisms and safe dosages through more clinical trials.

### 6.7 Vaccine

In addition to treatment and diagnosis, microbes can also be designed as vaccines for prevention (Table 3). Studies have shown that nanoparticle‐coated layers can enhance the dissemination of bacteria into the bloodstream, effectively improving antigen expression and antitumor immunity [156]. Bacteria activate antitumor immune responses through specific antigens. For example, Bacillus Calmette‐Guerin (BCG) is used for immunotherapy in bladder cancer [134]. Linfu Chen and colleagues developed a minimalistic, biomimetic nanovaccine by integrating highly immunostimulatory adjuvant cholesterol‐modified CpG oligonucleotides into autologously derived *F. nucleatum* membranes, which eliminates *F. nucleatum* without affecting the intratumoral and intestinal microbiota. It significantly enhances the efficacy of chemotherapy in *F. nucleatum* ‐infected CRC and reduces tumor metastasis [115]. The development of bacterial vaccines is expected to address challenges associated with nonselective bacterial eradication and the emergence of drug‐resistant strains. In addition to bacterial vaccines, the most commonly used vaccines currently are those related to viruses, such as HPV vaccines and hepatitis B vaccines. The application of vaccines often overlaps with strategies like engineered bacteria and OVs. For example, tumor‐specific bacterial vaccines designed based on the probiotic *E. coli* Nissle 1917 (a nonpathogenic strain) face similar challenges in clinical translation. In terms of safety, the long‐term behavior of engineered bacteria in the human body, risks of horizontal gene transfer, and potential excessive immune responses remain uncertain. Regarding efficacy, tumor heterogeneity is complex, and individual immune microenvironments vary significantly, meaning the response to vaccines can differ considerably depending on specific conditions. Moreover, orally administered vaccines are easily degraded by stomach acid and bile, which may compromise their effectiveness. In the future, addressing these issues may involve developing safer engineered bacteria, exploring novel carriers, optimizing delivery methods, and employing combination therapies.

### 6.8 Microbial‐Associated Metabolic Products

Chen et al. found that the supernatant of Bacillus toyonensis BV‐17 cultures could rapidly kill various tumor cell lines within minutes in vitro, resulting in cell membrane disruption, blebbing, and leakage of cytoplasmic content. The active molecule is hemolysin BL (HBL), and intratumoral injection of low‐dose HBL inhibits the growth of treated and untreated tumors in mice. It may act by damaging the cell membrane, forming vesicles, and causing leakage of cytoplasmic contents (Table 3) [116]. The results of this study indicate that HBL has antitumor activity and could be designed as a potential therapeutic agent targeting tumor cells in the future. Moreover, microbial metabolites such as inosine, short‐chain fatty acids (SCFAs), bile acids, and indoles are believed to be associated with the immunomodulation and therapeutic process of tumor cells (Table 3): (1) Inosine: Inosine enhances the immunogenicity of tumor cells and provides an alternative carbon source for CD8^+^T cells [117]. (2) SCFAs: SCFAs and immune checkpoint inhibitors (ICIs) have been recent research focuses. SCFAs can inhibit tumor cell differentiation, induce apoptosis, promote antitumor responses, and provide a carbon source for immune cells. In addition to some of the descriptions mentioned earlier in this review, butyric acid can also inhibit histone deacetylases (HDACs), and experiments have shown that selective HDAC8 inhibitors enhance the expression of CCL4, which is a key chemokine for T‐cell migration. This process can increase the density of CD8^+^T cells in mice with liver cancer [119]. Additionally, butyrate‐mediated inhibition of HDACs can upregulate the nuclear transcriptional regulator ID2. ID2 binds to the transcription factor E2A, relieving its inhibitory effect on IL‐12 receptor expression, thereby significantly upregulating the IL‐12R signaling pathway in CD8^+^T cells. This process increases the density and activation of CD8^+^T cells in the TIME [157]. Another study demonstrated that butyric acid could increase the expression of IFN‐γ and granzyme B in CD8^+^T cells and regulate glycolysis, the TCA cycle, and fatty acid oxidation (FAO) in antitumor effector cells to improve the efficiency of ICI [118]. (3) Indole and tryptophan: Indole, a product of tryptophan metabolism, has been shown to play a role in ICI therapies in several studies. In preclinical models of pancreatic cancer, Hezaveh et al. showed that indole activates the AhR activity, directing macrophage polarization, inhibiting inflammatory T‐cell infiltration, and promoting tumor growth [79]. Recently, however, Bender et al. have provided opposite results. In preclinical melanoma models, the study elucidates how the tryptophan metabolite I3A, produced by intratumoral *Lactobacillus reuteri* , promotes IFN production in a cAMP Response Element‐Binding Protein (CREB)‐dependent manner and enhances ICI therapy in advanced melanoma patients [108]. Additionally, providing a tryptophan‐enriched diet or intratumoral injection of I3A also generates a mimetic effect [158]. Similarly, *Lactobacillus gallinarum* ‐derived indole‐3‐carboxylic acid (3‐ICA) has recently been reported to boost anti‐PD1 efficacy in CRC [120]. These heterogeneous outcomes may arise from the fact that different indole derivatives can influence the TIME by acting on distinct immune cells, thereby exerting either inhibitory or promotive effects on tumors. Moreover, such effects are likely tumor type dependent, and further evidence is needed to substantiate these observations.

Consequently, the efficacy of microbial metabolites appears closely linked to tumor heterogeneity. The TME is dynamic, with significant variations in microbial composition across different tumor types, individuals, and even within the same tumor. Interactions between microbial metabolites and the resident ITM also play a contributing role. Additionally, the mechanisms of action of microbial metabolites remain poorly defined, and causal relationships require urgent investigation. The intricate network of interactions between microbial metabolites, immune cells, and tumor cells has yet to be fully elucidated, making precise modulation challenging. Some microbial metabolites may even exhibit dual effects. Therefore, selecting appropriate metabolites and applying them to suitable patient populations are crucial for ensuring therapeutic efficacy. Safety and regulatory considerations must also be addressed. Moving forward, more in depth research is needed on the precise selection, targeted delivery, and personalized combination therapies involving microbial metabolites.

Some researchers have also noted interactions between fungi and bacteria (Table 3). For instance, in head and neck cancer and GC, Candida is closely associated with Lactobacillus, and previous studies have reported interactions between Lactobacillus and Candida that can affect the pathogenicity of the latter. In GC, yeast often coexists with *H. pylori* [51]. This may also provide a potential therapeutic approach.

## 7 Future Research Directions

Current research has confirmed the association between intratumoral microbiota (ITM) and tumor progression, and the discovery and study of ITM may offer new perspectives for cancer treatment. However, further studies are needed to elucidate these relationships in greater detail, and translating research findings into safe and effective clinical therapeutic strategies still poses challenges. Firstly, whether the sampling of ITM is accurate and whether the analytical methods are reasonable may be an important factor affecting studies on the relationship between ITM and tumors. Microbiota, particularly ITM, exist in very low biomass. Any sample contamination can significantly impact the outcomes of microbial studies. Therefore, measures such as minimizing environmental contamination during sampling, randomizing sample selection, analyzing environmental microbiota in the surrounding sampling area, and including control groups in experiments may help reduce the risk of related contamination. Additionally, the abundance and diversity of microbiota vary depending on their sources. To obtain objective research results and prevent bias, various samples should be collected and analyzed, including primary tumor tissues, adjacent normal tissues, metastatic lesions, tumor‐associated malignant effusions, and body fluids from cancer patients. Currently, multiple methods for sequencing and analyzing ITM are applied in research, primarily including 16S rRNA sequencing, whole‐genome metagenomic sequencing, immunohistochemistry, immunofluorescence, proteomics, metabolomics, spatial transcriptomics, organoids, nanotechnology, and more. The diverse requirements of these techniques for sample sources can easily lead to heterogeneity bias due to variations in sample origins and also increase the difficulty of sample acquisition. Furthermore, most current studies are conducted in preclinical models and have not established robust causal relationships between ITM and tumors. The mechanisms by which ITM enter tumor cells remain incompletely understood. For some proposed mechanisms, research is limited to correlative evidence, lacking in‐depth analysis of the pathways involved. Moreover, the few clinical trials conducted to date have not achieved results comparable to those from preclinical studies, which represents a key challenge in translating findings into clinical applications. Utilizing appropriate and ideal models, such as patient‐derived tumor xenograft models, may facilitate the transition from preclinical to clinical research. And more longitudinal studies and clinical trials are needed to examine the dosage and safety of microbial therapies, and due to the presence of multiple factors such as age, gender, ethnicity, genetics, lifestyle habits, dietary patterns, and geographical differences, the requirements for clinical trials are correspondingly increased.

Additionally, in clinical applications, the precise regulation of microbiota should be enhanced, including optimizing targeted delivery and precision therapy, strengthening their colonization and survival within the tumor microenvironment (TME), and maintaining efficacy while reducing related adverse effects. Current research has explored various approaches in this regard, such as engineering delivery vectors and developing novel materials responsive to the TME. Furthermore, the TME is complex and dynamically changing. As previously mentioned, for example, microbial metabolites targeting AhR yielded different experimental outcomes in preclinical models of melanoma versus pancreatic cancer, indicating that the effects of microbiota‐related therapies are also influenced by specific tumor types or environmental contexts. ITM and the TME interact bidirectionally. Introducing or removing a particular microbiota in experimental studies may easily disrupt the balance of the native microbial community. Moreover, deeper and more comprehensive research is needed to determine whether certain effects—especially those involving nonspecific metabolic pathways—on tumor immunity are directly linked to specific microbiota.

Combination therapy with existing treatments represents a future direction for ITM‐based therapies. In‐depth studies are needed to determine the optimal timing and sequencing for modulating ITM to enhance the efficacy of radiotherapy, chemotherapy, and immunotherapy. For instance, whether microbial modulation should be applied before immune checkpoint inhibitors (ICIs) to precondition the tumor immune microenvironment (TIME), or whether engineered bacteria should be combined with ICIs to harness microbes as modulators for synergistic anti‐tumor effects. For personalized applications, it is essential to develop predictive models based on the patient's tumor type, ITM profile, genomic characteristics, and immune status. These models can guide the selection of the most suitable microbial modulation strategy—such as determining which engineered bacteria or phages to use, and which therapies to combine—thereby advancing true personalized precision medicine. Additionally, the safety and regulatory frameworks for tumor microbial therapies require continuous refinement. A rigorous biosafety assessment system for engineered microbiota must be established, covering their genetic stability in vivo, long‐term colonization potential, and clearance strategies, among other factors.

The focus of future research must shift from exploring correlations to intervening based on causality and achieving precise regulation. Key challenges for future translational application include how to target tumors specifically, how to selectively modulate particular microbiota, how to apply microbial therapies effectively, and how to screen patients who are most likely to benefit from such approaches. With advances in technology, the establishment of standards, and the growing body of research on the microbiota in human cancers, it is believed that therapies based on ITM will offer more options for cancer patients in the future.

## 8 Conclusion

Overall, this review discusses the roles and distributions of ITM, with examples including bacteria and fungi, and their interactions with the TIME. Given the significant role of ITM in tumor immunity, it may serve as a potential target for enhancing immunotherapy. Furthermore, ITM could be used as diagnostic tools, prognostic indicators, and therapeutic targets. We hope our review can provide some ideas for further innovation and clinical applications in the field of ITM.

## Author Contributions

**Fengxue Li:** writing – original draft (equal), writing – review and editing (equal). **Lili Qiao:** writing – original draft (equal), writing – review and editing (equal). **Xinquan Liang:** writing – review and editing (equal). **Yingying Zhang:** writing – review and editing (equal). **Ning Liang:** writing – review and editing (equal). **Jian Xie:** writing – review and editing (equal). **Guodong Deng:** writing – review and editing (equal). **Yuying Hao:** writing – review and editing (equal). **Pingping Hu:** writing – review and editing (equal). **Xue Wu:** writing – review and editing (supporting). **Fangjie Ding:** writing – review and editing (supporting). **Can Feng:** writing – review and editing (supporting). **Yiming Mu:** writing – review and editing (supporting). **Jiandong Zhang:** writing – review and editing (equal).

## Funding

This work was supported by the Natural Science Foundation of Shandong Province, China (ZR2021LSW023, ZR2021QH356).

## Ethics Statement

The authors have nothing to report.

## Consent

The authors have nothing to report.

## Conflicts of Interest

The authors declare no conflicts of interest.

## Data Availability

The authors have nothing to report.
